# Global Protein Profiling in Processed Immunohistochemistry Tissue Sections

**DOI:** 10.3390/ijms241411308

**Published:** 2023-07-11

**Authors:** Simone Venz, Viola von Bohlen und Halbach, Christian Hentschker, Heike Junker, Andreas Walter Kuss, Thomas Sura, Elke Krüger, Uwe Völker, Oliver von Bohlen und Halbach, Lars Riff Jensen, Elke Hammer

**Affiliations:** 1 Institute of Medical Biochemistry and Molecular Biology, University Medicine Greifswald, 17475 Greifswald, Germany; heike.junker@med.uni-greifswald.de (H.J.); elke.krueger@med.uni-greifswald.de (E.K.); 2 Institute of Anatomy and Cell Biology, University Medicine Greifswald, 17489 Greifswald, Germany; viola.bohlenundhalbach@uni-greifswald.de (V.v.B.u.H.); oliver.vonbohlen@uni-greifswald.de (O.v.B.u.H.); 3 Interfaculty Institute of Genetics and Functional Genomics, University Medicine Greifswald, 17475 Greifswald, Germany; hentschkec@uni-greifswald.de (C.H.); kussa@uni-greifswald.de (A.W.K.); thomas.sura@uni-greifswald.de (T.S.); voelker@uni-greifswald.de (U.V.); jensenl@uni-greifswald.de (L.R.J.)

**Keywords:** protein preparation, formalin fixed and embedded brain sample, mass spectrometry, SDS-SP3 protocol

## Abstract

Tissue sections, which are widely used in research and diagnostic laboratories and have already been examined by immunohistochemistry (IHC), may subsequently provide a resource for proteomic studies, even though only small amount of protein is available. Therefore, we established a workflow for tandem mass spectrometry-based protein profiling of IHC specimens and characterized defined brain area sections. We investigated the CA1 region of the hippocampus dissected from brain slices of adult C57BL/6J mice. The workflow contains detailed information on sample preparation from brain slices, including removal of antibodies and cover matrices, dissection of region(s) of interest, protein extraction and digestion, mass spectrometry measurement, and data analysis. The Gene Ontology (GO) knowledge base was used for further annotation. Literature searches and Gene Ontology annotation of the detected proteins verify the applicability of this method for global protein profiling using formalin-fixed and embedded material and previously used IHC slides.

## 1. Introduction

Due to the great potential for the screening for disease-specific and prognostic signatures, analytical methods for studying protein composition in tissue have developed rapidly in recent years. All steps of sample preparation as well as the technical aspects of analytical instruments have to be optimized. In the past, most of the analyses were performed on freshly frozen material. However, the collection of material snap-frozen in li-quid nitrogen directly in the operation theater is difficult to implement into hospital routine and limits thereby the number of available tissue samples. In contrast, formalin-fixed and embedded (FFE) tissue sections for histopathological diagnosis that can be stored for years are prepared from many different patients. These samples constitute a huge repo-sitory with great potential for detection of possible biomarkers. This is especially important for rare diseases, presenting in low numbers per location and period [1,2,3]. The use of samples prepared through the fixation of fresh tissue samples with a formalin-based solution, is interesting, as the fixative also stabilizes the samples and preserves their cellular composition, allowing for easy storage, transportation, and handling, as well as a time-independent analysis [4,5,6,7,8,9]. Formalin-fixed material embedded in either cover matrices (FFE) or paraffin (FFPE), is extensively applied in various diagnostic fields of medicine, such as disease determination, immunohistochemical staining, or quantification of biomolecules [10,11,12]. The identification of proteins by liquid chromatography–mass spectrometry (LC-MS/MS) using FFPE samples has gained considerable attention, but remains a challenge [13]. Cross-linking processes, fixation, and denaturation of the samples and their influence on the analysis must be taken into account. With various approaches, quantitative analyses can also be performed from such tissue samples using mass spectrometry. To selectively and sensitively detect biomolecules, specific sample preparation and additional enrichment methods have been developed and successfully applied in combination with LC-MS/MS. Achieving optimal results in the extraction of proteins from tissues requires careful consideration of the composition of the lysis buffers used, with many containing SDS. To obtain optimal outcomes, it is necessary to conduct additional processing that is compatible with mass spectrometry, such as FASP (filter-aided sample preparation) or SP3 (single pot, solid phase, sample preparation) protocols [13,14]. In addition, various enrichment methods such as LBA (hybrid ligand-binding assays) or antipeptide antibody immunocapture followed by LC-MS/MS have been developed in recent years to target specific proteins [15,16,17,18]. However, the focus on proteins of interest limits the potential for much broader analyses of the samples.

Several research studies use laser microdissection as noncontact methods to isolate tissue areas or cells from tissue slices [19]. Dedicated slides exist for laser microdissection devices, using various technologies and different microscopy imaging techniques [20,21,22]. However, other slide types are routinely used in histopathology, and therefore such preparations have not been considered so far for micro- or macrodissection and subsequent proteomics analysis. Therefore, our aim was to harness this valuable specimen resource for secondary use for proteomics. Our method is applicable not only for clinical specimens but also for animal samples with low material input. As a result, it is possible to directly correlate the protein profile with the morphological pictures. Here, we introduce a robust workflow for investigations on tissue sections already used for histopathology using macrodissections from different brain areas of mice as an example.

## 2. Results

### 2.1. Protein Extraction Protocols for Proteomics

**Figure 1:**
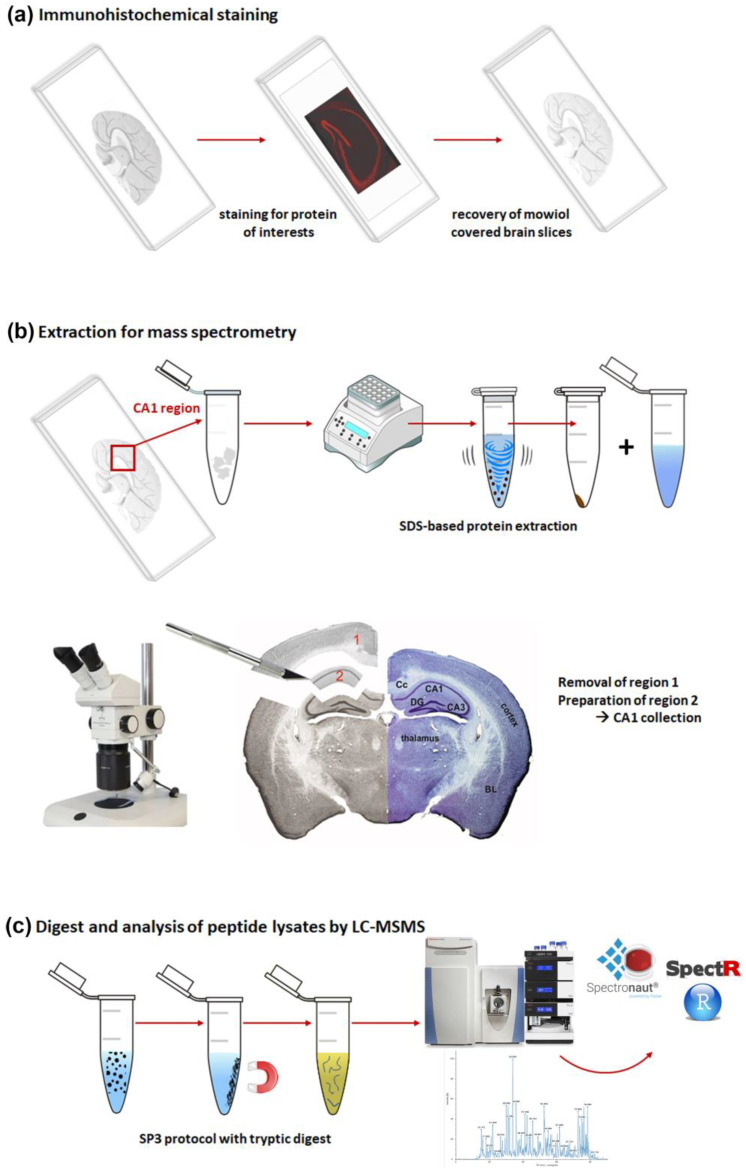
Schematic overview of the protocol used to prepare FFE samples for mass spectrometric analysis. (**a**) Starting point—embedded brain sections on glass slides; immunohistochemical staining was performed for different proteins of interest; (**b**) extraction procedure—dissection of target region, transfer into tubes and SDS-based protein extraction; microscopic picture of the extracted region CA1 = region 2 with counterstaining with cresyl violet (right); (**c**) protein digest using SP3 protocol with subsequent mass spectrometric data recording and database search. Abbreviations: BL: basolateral amygdala; Cc: corpus callosum; DG: dentate gyrus.

The embedded tissue sections were processed according to the scheme shown in Figure 1. The brain slices on slides already used for the immunohistochemical studies were first treated with xylene in order to gently remove the coverslip with a pair of forceps without affecting the section. Subsequent rehydration made the tissue accessible for further processing (Figure 1a). Here, we chose to investigate the CA1 region of the hippocampus and located it in the tissue slice by binocular inspection. The defined area was excised from the 30 µm-thick section representing approximately 0.1–0.2 mm^3^ of material. Pooled samples of eight parallel sections per mouse were used for further investigations (Figure 1b) taking into consideration the small CA1 area per slice. The detailed steps of sample extraction from the slides are shown in Supplementary Figure S1.

In order to evaluate complexity and region specificity of the identified CA1 proteome, we analyzed whole brain lysates from mice of the same strain and age. For extraction of proteins, we used SDS-containing buffers. To avoid possible interference of the detergent with the mass spectrometric analysis, sample preparation was performed with the SP3 protocol, including proteolytic digestion of the proteins and peptide purification on magnetic beads (Figure 1c). In contrast to whole brain lysates, a determination of protein or peptide amounts in the extracts of the CA1 region from slides was not possible. However, standardized analysis of protein samples for meaningful comparison represents an important challenge in proteomic research. Therefore, the MS1 signal intensity from a preliminary LC-MS/MS run using only 1 µL (out of 12 µL) of each sample was used to determine the injection volume that would ensure comparable peptide loading.

### 2.2. Protein Pattern of CA1 Region

**Figure 2:**
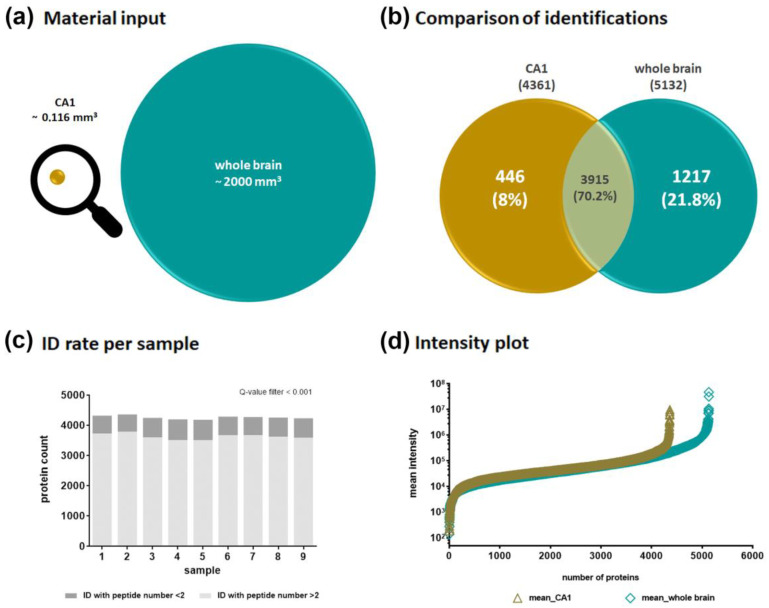
Comparison of protein identifications. (**a**) Starting point—used volumes of material input; (**b**) protein group identifications—comparison between CA1 region and whole brain extracts; (**c**) identification rate per sample in CA1 region samples; (**d**) protein intensity plot—triangle symbols based on all identified protein groups for the CA1 region extract, upright square symbols based on all identified proteins for the whole brain extract.

Using similar extraction and sample preparation procedures as well as the same MS and data analysis method for the whole brain, both proteomes could be qualitatively compared (Figure 2). Although very little material from the CA1 region was available (Figure 2a), it was possible to generate a comprehensive protein profile. With the DirectDIA algorithm implemented in Spectronaut (Biognosys, Zurich, Switzerland), 4361 different protein groups were identified for the CA1 region. We did not detect any significant level of the primary antibodies used, which could have impaired the mass spectrometric proteome analysis. In the whole brain lysates representing the whole range of different brain regions, 5132 protein groups were identified. The overlay of both proteomes consisted of 3915 proteins (Figure 2b) and indicated that a high number of proteins are common in different brain regions, as reported by Korovesi et al. [23]. Beside this, we were able to additionally identify 446 proteins exclusively in the extracted CA1 region. This emphasizes the advantage of the method used in relation to the detection of region-specific proteins. In Figure 2c, the protein identification rates per CA1 sample are reported to corroborate the reproducibility of the method. In sum, 4200 to 4360 proteins were identified per sample. The protein identifications are based on at least two peptides for approximately 84–87% of the proteins in the replicates, and another 13–16% were able to be identified on the basis of one peptide. The intensity plots for the CA1 samples as well as for the whole brain samples show a similar distribution across the entire intensity range sufficient for MS identification and relative quantification (Figure 2d).

Supplementary Table S1 provides a summary of all proteins identified in the samples of the CA1 region, and the 446 proteins exclusively found in this area are labeled as such. Of those, 27 CA1 proteins (Table 1) are particularly important for the functionality of the CA1 region, and their protein intensities are shown in Supplementary Figure S2.

Several of the proteins specifically detected in the CA1 region are involved in neuronal activity, processes attributed to neuronal and synaptic plasticity, and hippocampus-dependent learning and memory functions (Supplementary Table S2).

### 2.3. Functional Enrichment Analysis of the Proteins Identified in the CA1 Region

**Figure 3:**
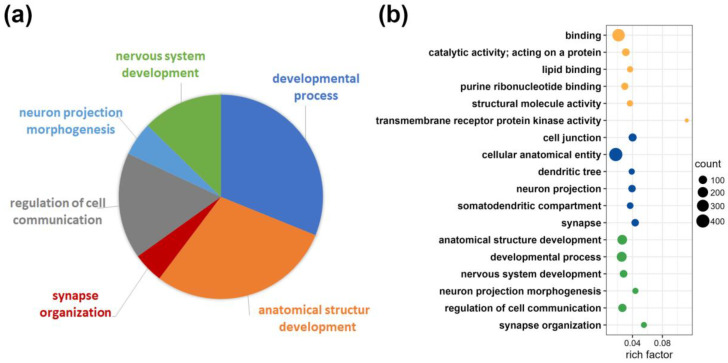
Functional enrichment analysis with g:Profiler for protein groups exclusively identified in the CA1 region. (**a**) Pie chart of protein portions assigned to the GO term “biological process”. (**b**) Rich factor representing detailed information on the categories identified by the functional enrichment analysis; ratio of differentially expressed protein number annotated in the specific pathway term to all protein numbers annotated within the pathway; yellow dots for Gene Ontology term “molecular function”; blue dots for Gene Ontology term “cellular component”; green dots for Gene Ontology term “biological process”; the size of the circles corresponds to the number of protein groups represented (counts).

To estimate the specificity of the analysis, we subjected the data to a functional profiling search. With g:Profiler (https://biit.cs.ut.ee/gprofiler/gost, accessed on 17 April 2023), [24]), a functional enrichment analysis was performed with the 446 protein groups exclusively identified for the CA1 region (see Figure 2b). The terms statistically significantly enriched using multiple testing correction by default and tailor-made algorithm using g:SCS for reducing significance scores [25] are shown in Figure 3. Detailed information is given for cellular component, biological process and molecular function. The assignment of proteins from the CA1 region to specific GO terms such as anatomical structure development, synapse or synapse organization, nervous system development or neuron projection underline the importance of the findings for functions in this specific brain region (Figure 3a,b and Supplementary Table S3).

### 2.4. Ingenuity Pathway Analysis

In addition, the 446 proteins that were exclusively found in the CA1 region extracts (Supplementary Table S1) were used to generate a picture of the cellular regulatory processes. Among other categories, the proteins belong to transporters (30), enzymes (78), G-protein-coupled receptors (5), peptidases (9), transcription regulators (28), ion channels (14), kinases (25), transmembrane receptors (5), growth factors (2) and phosphatases (7).

The top three canonical pathways are listed in Table 2. In these pathways, also proteins important for the specific CA1 functionality (see Table 1) are found (marked in bold red).

Different protein networks were built by IPA describing “top diseases and function”. Networks 1, 2, and 3 showed high overlap in the assigned categories—“cell death and survival, neurological disease, organismal injury and abnormalities” (network 1 and 2)—and additional “free radical scavenging” instead of “neurological disease” in network 3, but included different molecules (Table 3). For the network construction, 35 proteins were defined within the parameter set, of which 16 proteins belonging to networks 1 and 15 proteins belonging to networks 2 and 3 were found in this study. The graphical representation is given in Supplementary Figure S4. Additional information for specific categories linked to disease and functional annotation are given in Supplementary Table S4.

### 2.5. Comparison of Tissue-Derived CA1 Protein Patterns to Published Data Sets

**Figure 4:**
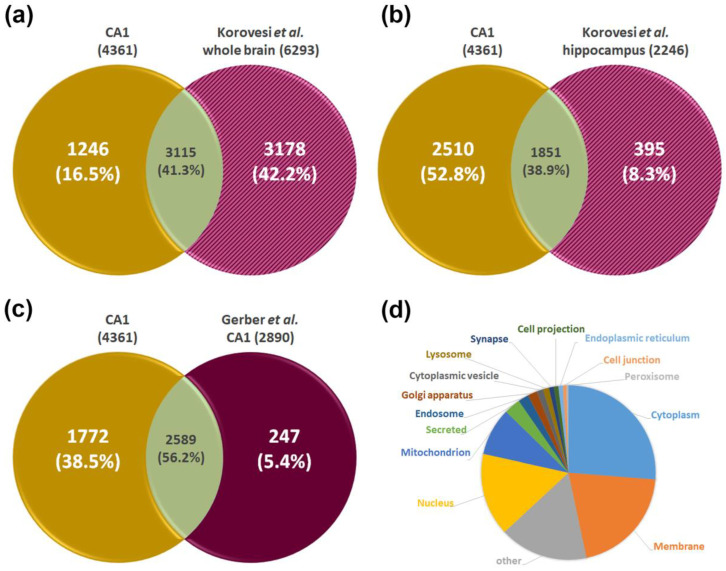
Comparative analysis of the CA1 proteome with published datasets. The CA1 proteome data are compared to the murine data set published by Korovesi et al. [23]. (**a**) Comparison to cumulative data of 7 brain regions mentioned as “whole brain”. (**b**) Comparison to the proteome reported for the hippocampus region; (**c**) comparison of CA1 proteome to that reported by Gerber et al. [26] derived from CA1 region dissected from fresh murine brain; (**d**) subcellular localization of the 1772 exclusively identified proteins (based on UniProt Knowledgebase).

In the final step, we compared our data with published literature. A comprehensive map of the proteomes of different brain regions obtained from fresh organs was published by Korovesi et al. [23] describing 6293 individual gene products identified across individual brain regions in wild-type mice. Although a CA1-specific proteome was not analyzed, several other regions, including cerebral cortex, olfactory bulb, hypothalamus, midbrain, and cerebellum, and the hippocampus as well as medulla with hippocampus were studied. Of the 4361 proteins found in this study for CA1, 3115 could be assigned to fit the literature data, being common proteins without any specific classification (Figure 4a). The detailed comparison of the hippocampus data with the data obtained for the CA1 region as part of the hippocampus in this study showed an overlap of 1851 proteins (Figure 4b), but also 1246 proteins exclusively found in the CA1 proteome data set (Figure 4a).

A proteome study with focus on the murine CA regions was performed by Gerber et al. [26]. The analysis of the CA1 region dissected from fresh brain tissue revealed 2890 different proteins. Due to the obviously higher sensitivity of our workflow, in our study, a significantly higher number of proteins was identified (Figure 4c). The additionally identified 1772 proteins were classified according to their subcellular localization using information from the UniProt Knowledgebase (Figure 4d), and the results show on which compartments additional insights were gained in the current study.

## 3. Materials and Methods

### 3.1. Mice

The nine C57BL/6J mice used for this study were between 22 and 25 weeks old at the time of testing. The *Landesamt für Landwirtschaft, Lebensmittelsicherheit und Fischerei* (LALLF, Mecklenburg-Vorpommern, Germany) approved the animal studies. The investigation conforms to the *Guide for the Care and Use of Laboratory Animals* published by the US National Institutes of Health (NIH publication 85-23, revised 1985), as well as the current version of German Law on the Protection of Animals.

### 3.2. Preparation of Embedded Slices

Mice were euthanized and transcardially perfused with phosphate-buffered saline (PBS) and afterwards with 4% paraformaldehyde (PFA) in PBS. The brains were removed and postfixed in PFA for at least 3 days. Coronal sections (30 µm thick) from extracted fixed brain (between Bregma −1.46 and −2.5) were sliced using a vibratome (VT 1000S, Leica, Wetzlar, Germany). Sections were applied on glass slides and then air-dried. For antigen retrieval, the sections were transferred for 20 min into a microwave (800 W) in the presence of sodium citrate buffer (pH 6.0). After rinsing, sections were incubated in PBS (with 3% serum and 0.1% Triton-X-100) containing α/ß tubulin antibodies (1:100, polyclonal rabbit antibody; Cell Signaling Technology, Danvers, MA, USA) overnight. Next, the sections were rinsed and then transferred into a solution containing Cy3-conjugated goat anti-rabbit IgG (1:400; Vector Labs. USA) in the presence of 0.1% Triton-X-100 and 3% serum (dissolved in PBS). After further rinsing, sections were counterstained with DAPI (0.1 µg/mL) and embedded in Mowiol (Sigma-Aldrich, Taufkirchen, Germany). The sections were analyzed using a microscope fitted for fluorescence. After analysis, sections were stored at 4 °C in the dark for several months.

### 3.3. Preparation of CA1 Region Samples for Mass Spectrometry

Samples already used for immunofluorescence staining were processed for mass spectrometric analysis. The embedded sections were transferred into a xylene bath for several days in order to detach the coverslip. After removing the coverslip, the sections located on the slides were rehydrated by submersion in ethanol diluted in distilled water with a descending alcohol series (96% for 1 h; 70%, 50%, 20%, each for 5 min). CA1 areas were excised and pooled from eight individual subsequent slices per animal (around Bregma −2.06 to −2.30) using a scalpel and binocular (Askania SMT4, Mikroskop Technik, Rathenow, Germany). The collected tissue was stored in 20% ethanol (for details, see Supplementary Figure S1).

Dissected tissues available from 9 mice samples were incubated in 40 µL extraction buffer (4% SDS, 100 mM dithiothreitol (DTT) in 100 mM Tris-HCl pH 8.0) at 4 °C for 5 min. Protein extraction was performed with shaking for 20 min at 95 °C followed by 2 h at 80 °C with inversion of samples every 10 min. After short cooling on ice, centrifugation was performed for 15 min at 14,000× *g* (4 °C) and the protein containing supernatant collected. For MS analysis, peptide lysates were prepared following the adapted SP3 protocol using 1:1 hydrophobic and hydrophilic Sera-Mag SpeedBeadsTM (Cytiva, Freiburg, Germany). Samples were diluted with 40 µL 20 mM Tris-HCl and subjected to reduction (2.5 mM DTT at 37 °C, 30 min) and alkylation (10 mM iodoacetamide (IAA) at 37 °C, 15 min). Alkylation reaction was quenched by addition of 2.5 mM DTT. Protein digestion with trypsin (enzyme to protein ratio 1:25) and purification of peptides were performed according to Blankenburg et al. [27]. In detail, protein lysates were mixed with SP3 beads (12 µL) and the suspension was diluted with 100% acetonitrile to a final acetonitrile concentration of 70% (*v*/*v*), mixed well and incubated at 1400 rpm and RT for 18 min in a thermoshaker (TS 100, PEQLAB Biotechnologie GmbH, Erlangen, Germany). After an incubation for 2 min on the DynaMag™-2 Magnet, the supernatant was discarded. Beads were washed two times in 180 µL 70% (*v*/*v*) ethanol and two times in 180 µL 100% acetonitrile by resuspension and repeated incubation on the magnet for 2 min. Before digestion overnight at 37 °C, the beads were dried for 5 min and resuspended in 20 mM ammonium bicarbonate buffer subsequently. The digestion was stopped by addition of 100% acetonitrile to a final acetonitrile concentration of 95% (*v*/*v*). The beads were washed twice with 180 µL acetonitrile as described above and dried for 5 min. After addition of 10 µL 2% (*v*/*v*) DMSO and sufficient resuspension, peptide elution was supported by incubation in an ultrasonic bath for 3 min. Beads were separated on the magnet for 2 min. The supernatant (peptide solution) was transferred to a new micro-reaction tube. The second incubation on the magnet removed all remaining beads. The peptide solution was transferred to a vial with micro-insert (VWR) and diluted with 10 µL 2x MS-buffer (4% acetonitrile, 0.2% acetic acid). Samples were stored at −80 °C until measurement.

### 3.4. Preparation of Whole Brain Samples for Mass Spectrometry

Whole brain tissue was extracted and immediately flash frozen in liquid nitrogen. The tissue was homogenized in a microdismembrator (Sartorius, Göttingen, Germany) for 2 min at 2600 rpm. For protein extraction, approximately 20 mg of the resulting tissue powder was mixed with 200 µL extraction buffer containing 5% SDS (20 mM HEPES pH 7.5; 1× cOmplete; 1× PhosSTOP, both from Roche, Basel, Switzerland) and incubated for 5 min at 95 °C. After dilution of the SDS concentration to 1%, 25 U of universal nuclease (Pierce/Thermo, Rockford, IL, USA) was added and the lysates incubated at 37 °C for 15 min to degrade polynucleotides. After centrifugation for 20 min at 17,000× *g*, supernatant was collected and protein concentration determined using a micro-BCA assay (Pierce).

Five micrograms of protein were reduced by adding tris(2-carboxymethyl)phosphine (5 mM) and incubated at 60 °C for 30 min followed by alkylation of cysteines with IAA (10 mM, 37 °C, 20 min). Subsequent protein digestion by LysC (1:100 protease:protein) for 3 h in 50 mM TRIS-HCl pH 8 at 37 °C and trypsin (1:25 protease:protein) for 15 h at 37 °C and peptide purification was carried out using the adapted SP3 protocol [27] with SP3 beads at a bead:protein ratio of 10:1.

### 3.5. Mass Spectrometric Analysis

Peptides were analyzed on a QExactive HF Hybrid Quadrupole-Orbitrap Mass Spectrometer (Thermo Scientific, Bremen, Germany) coupled to a nano-LC system (Ultimate 3000, Thermo Scientific). Mass spectra were recorded in data-independent acquisition (DIA) mode and data were analyzed via the DirectDIA algorithm implemented in Spectronaut (Biognosys, Zurich, Switzerland) using a Uniprot/SwissProt database (v. 2022_04) for mouse sequences. Search criteria for peptide/protein identification and intensity extraction were fixed to: minimal peptide length = 7; 2 missed cleavages; carbamidomethy-lation as fixed modification and acetylation (protein N-term)/oxidation (M) as variable modifications; and quantity type = area. Filter criteria (q-value) applied for significant peptide and protein identification were: ions < 0.001; peptides < 0.01 and proteins < 0.01 with an FDR of <0.01 (Supplementary Table S5). For protein group annotation, the internal algorithm IDpicker was used.

### 3.6. Bioinformatical Data Analysis and Statistics

The list of proteins was further analyzed by exploring expression patterns of the corresponding coding genes, protein functions and putative protein interactions in brain tissue. Functional enrichment analysis was performed using the open-source webserver g:Profiler (e107_eg54_p17_bf42210) [24] and Ingenuity Pathway Analysis (IPA) (release 46901286, QIAGEN Bioinformatics, Venlo, The Netherlands). Furthermore, IPA was used to construct the shortest hypothetical pathway networks, showing the most relevant direct and indirect interactions of the regulated proteins and the proteins predicted to be involved in the interactions. For data presentation, GraphPad Prism software v. 6.07 was used (GraphPad Prism software Inc., La Jolla, CA, USA). Data are presented as means ± SEM. RStudio 2022.12.0 (Posit Software, PBC, Boston, MA, USA) with the implemented package tidyverse 2.0.0 was used to display the functional enrichment data (rich factor). An interactive tool for comparing lists was used to create Venn diagrams (https://bioinfogp.cnb.csic.es/tools/venny/index.html, accessed on 13 April 2023 [28]).

## 4. Conclusions

The use of FFE and FFPE samples for mass spectrometric analysis has been extended to many tissue types and many research questions in recent years. Most frequently, the procedures have been used for cancer samples and have been successfully applied for colon carcinoma [29], thyroid cancer, or lung tumors [30,31,32]. However, there is a high potential for the study of material of patients suffering from rare diseases by reusing slides already used for histological inspection by pathologists from various hospitals across the world. In our study, we showed that the processing of FFE samples using SDS-based SP3 protocols for protein identification and relative quantification is even possible for already immunohistochemically used and fluorescently stained tissue sections. This finding underlines the results of Griesser et al. [4]. The group compared different reprocessing protocols for laser-capture microdissected brain tissue FFPE samples and revealed best protein yields and protein coverages with the SDS-SP3 protocol.

We used the workflow for long-preserved FFE samples already used for IHC staining and a manual macrodissection of a region of interest. With over 4300 identified protein groups from only partial areas of an FFE slice, the protocol allowed comparable protein identification and quantification rates, as reported recently for manual and half-automated sample preparation [30,33]. The correlation of protein patterns and functional relationships with already known information, e.g., obtained from immunohistochemical investigations, showed a high level of accuracy. Even very small amounts of protein are sufficient for this type of processing, which makes the method also attractive for limited embedded material of murine organs and specific regions of interest.

Because formalin-fixed tissue can be readily archived for decades with the appropriate patient data for retrospective evaluation [34], such tissue represents an available resource of clinical information that is essential for retrospective studies. Accurate profiling of their protein content would greatly enhance the potential for discovery and validation of clinically relevant features.

## Figures and Tables

**Table 1:** Selection of 27 proteins identified exclusively in the CA1 region with particular importance for area-specific functionality. Information was obtained from UniProt and PubMed search.

Entry Name	Protein Name	Gene	Function/ Involvement in *	Mean Intensity; Range 180–9,700,000
APOH_MOUSE	Beta-2-glycoprotein 1	*Apoh*	cognitive decline	13,058
CD38_MOUSE	ADP-ribosyl cyclase/cyclic ADP-ribose hydrolase 1	*Cd38*	dendritic organization long-term synaptic depression	59,130
CRHBP_MOUSE	Corticotropin-releasing factor-binding protein	*Crhbp*	stress susceptibility	30,011
CSMD3_MOUSE	CUB and sushi domain-containing protein 3	*Csmd3*	dendrite development	21,276
DOC2B_MOUSE	Double C2-like domain-containing protein beta	*Doc2b*	neuronal activity synaptic release	18,311
DOP2_MOUSE	Protein dopey-2	*Dop1b*	mental retardation	24,725
EPHA5_MOUSE	Ephrin type-A receptor 5	*Epha5*	synaptic plasticity	72,835
FGF13_MOUSE	Fibroblast growth factor 13	*Fgf13*	synaptic excitatory-inhibitory imbalance	32,178
FBCD1_MOUSE	Fibrinogen C domain-containing protein 1	*Fibcd1*	neurodevelopmental disorder	14,255
FLRT2_MOUSE	Leucine-rich repeat transmembrane protein FLRT2	*Flrt2*	synaptic plasticity spatial memory	35,696
FUT8_MOUSE	Alpha-(1,6)-fucosyltransferase	*Fut8*	hippocampal Long Term Potentiation	5973
KIRR3_MOUSE	Kin of IRRE-like protein 3	*Kirrel3*	intellectual disability	5782
LEG3_MOUSE	Galectin-3	*Lgals3*	hippocampal formation	13,470
LRFN2_MOUSE	Leucine-rich repeat and fibronectin type-III domain-containing protein 2	*Lrfn2*	synaptic adhesion synaptic plasticity	18,303
MDGA1_MOUSE	MAM domain-containing glycosyl-phosphatidylinositol anchor protein 1	*Mdga1*	synapse inhibition	33,520
MK_MOUSE	Midkine	*Mdk*	memory	14,722
MT3_MOUSE	Metallothionein-3	*Mt3*	apoptosis of neurons	13,294
MYD88_MOUSE	Myeloid differentiation primary response protein MyD88	*Myd88*	signal transduction neuronal activity	10,861
NTNG2_MOUSE	Netrin-G2	*Ntng2*	axon differentiation	25,387
PK3CG_MOUSE	Phosphatidylinositol 4	*Pik3cg*	long-term depression cognitive impairments	7427
KS6B1_MOUSE	Ribosomal protein S6 kinase b1	*Rps6kb1*	developing and mature of neuronal cells	29,356
RTN4R_MOUSE	Reticulon-4 receptor	*Rtn4r*	aging and cognitive decline	48,793
SORC3_MOUSE	VPS10 domain-containing receptor SorCS3	*Sorcs3*	postsynaptic modulation of synaptic depression	49,851
TAFA5_MOUSE	Chemokine-like protein TAFA-5	*Tafa5*	spatial memory	31,477
ITF2_MOUSE	Transcription factor 4	*Tcf4*	dendritic spine density	19,181
TRPC4_MOUSE	Short transient receptor potential channel 4	*Trpc4*	hippocampal synaptic transmission	30,792
ZEB2_MOUSE	Zinc finger E-box-binding homeobox 2	*Zeb2*	neuronal development	21,158

* References are provided in the Supplementary material.

**Table 2:** Assignment of proteins identified exclusively in extracts of the CA1 region to canonical pathways using ingenuity pathway analysis. The top 3 canonical pathways resulting from the analysis of the 446 exclusively found proteins are given. The overlap indicates the number of molecules detected in relation to the molecules assigned to the overall pathway (ratio is given as rich factor). All proteins matched to the pathway are listed as “molecules in dataset”. Proteins highlighted in bold red belong to the 27 proteins of interest listed in Table 1. A network of interactions is provided as Supplementary Figure S3.

Canonical Pathway	*p*-Value	Overlap Molecules in Dataset/Number of Molecules Whole Pathway	Rich Factor	Molecules in Dataset
G Beta Gamma Signaling	3.90 × 10^−5^	12/127	0.094	ARHGEF6, CACNB2, CACNG4, GNA14, GNB3, GNG5, GNG10, KCNJ6, KCNJ9, **PIK3CG**, PRKCI, PRKCZ
Opioid Signaling Pathway	8.09 × 10^−5^	18/276	0.065	CACNB2, CACNG4, GNA14, GNB3, GNG5, GNG10, GRK3, KCNJ6, KCNJ9, **PIK3CG**, PRKCI, PRKCZ, RAC3, RGS17, **RPS6KB1**, RYR1, RYR3, **TCF4**
Axonal Guidance Signaling	3.91 × 10^−4^	24/283	0.085	ARHGEF6, **EPHA5**, EPHA6, EPHA7, EPHB1, EPHB6, FZD7, GNA14, GNB3, GNG5, GNG10, HERC2, MMP17, NRP2, NTNG1, NTNG2, **PIK3CG**, PRKCI, PRKCZ, RAC3, ROBO1, **RTN4R**, SLIT1, UNC5C

**Table 3:** IPA network information. All proteins considered in the network are listed. Proteins highlighted in bold are part of the CA1-specific proteins. Proteins highlighted in bold red belong to proteins with particular importance for brain region CA1 functionality listed in Table 1.

ID	Molecules in Network	Score	Focus Molecules	Top Diseases and Functions
1	ADGRF5, **ADRM1**, APOL2, APP, CERK, CNRIP1, **EPHB6**, **FGF13**, GOLIM4, HDAC4, ITGB1, JUN, KCNAB3, **KCNJ6**, **KCNJ9**, **LAMB1**, **Map3k7**, MECP2, NACC2, **NCOR1**, NEXN, NF2, **NYAP1**, **PDGFRB**, PREX2, RAC1, **ROBO1**, **SH3RF1**, **SIDT1**, **SLC35F6**, **TBC1D2B**, TBC1D9B, **TCF20**, TSPAN9, UBQLN2	17	16	Cell Death and Survival, Neurological Disease, Organismal Injury and Abnormalities
2	**Abcb1b**, ACTB, **ACTG1**, APP, **ATP12A**, **Atp5e**, BACE1, CAT, DPYSL2, **EIF5A2**, EOMES, **FZD7**, GAP43, GFAP, GRM5, **H2AC7**, **HECW1**, **Ly6a** (includes others), MAPK14, MEF2C, **MRTFA**, **Mt3**, NTRK2, PLA2G4A, PSEN1, PTGER2, PURA, RELA, **SH3PXD2A**, SLC1A2, **SMPD2**, SNAP25, **SNAPIN**, **TCF4**, VAMP2	15	15	Cell Death and Survival, Neurological Disease, Organismal Injury and Abnormalities
3	**ARR3**, AURKA, BAD, BCL2L1, CAST, CDK5, DLGAP3, FTH1, **GABRD**, GNAS, **GNG10**, **GNG5**, **GRK3**, **Gstm6**, **HK2**, HMOX1, HSPA5, MAPK14, **MIB1**, Nefm, OPRK1, **P2RX7**, PDPK1, **PRKCI**, **RPS6KB1**, **RYR1**, SLC11A2, SLC40A1, SOD1, **SRXN1**, TFRC, THRB, **Tmsb4x** (includes others), **UTS2**, VIP	15	15	Cell Death and Survival, Free Radical Scavenging, Organismal Injury and Abnormalities

## Data Availability

Data are available on request subject to restrictions. The data presented in this study are available on request from the corresponding author (S.V. or E.H.).

## Supplementary Materials

The following material is available online at https://www.mdpi.com/article/10.3390/ijms241411308/s1.
