# Supplementary material for: Global Protein Profiling in Processed Immunohistochemistry Tissue Sections
Source: Int J Mol Sci. 2023 Jul 11;24(14):11308. doi: 10.3390/ijms241411308 (PMC10379013; doi:10.3390/ijms241411308)

Supplementary material

Table S1

Protein identifications by mass spectrometry for CA1 extract and whole brain extract

see separate Excel Table

Table S2

Biological functions of the selection of 27 hippocampal proteins exclusively in the CA1 region.

Information was obtained from UniProt.

Entry name	Protein name	Gene name	GO_Biol function
APOH_MOUSE	Beta-2-glycoprotein 1	Apoh	animal organ regeneration [GO:0031100]; blood coagulation [GO:0007596]; blood coagulation, intrinsic pathway [GO:0007597]; negative regulation of angiogenesis [GO:0016525]; negative regulation of blood coagulation [GO:0030195]; negative regulation of endothelial cell migration [GO:0010596]; negative regulation of endothelial cell proliferation [GO:0001937]; negative regulation of fibrinolysis [GO:0051918]; negative regulation of myeloid cell apoptotic process [GO:0033033]; negative regulation of respiratory burst [GO:0060268]; negative regulation of smooth muscle cell apoptotic process [GO:0034392]; plasminogen activation [GO:0031639]; positive regulation of lipoprotein lipase activity [GO:0051006]; positive regulation of triglyceride catabolic process [GO:0010898]; regulation of blood coagulation [GO:0030193]; regulation of fibrinolysis [GO:0051917]; triglyceride metabolic process [GO:0006641]; triglyceride transport [GO:0034197]
CD38_MOUSE	ADP-ribosyl cyclase/cyclic ADP-ribose hydrolase 1	Cd38	artery smooth muscle contraction [GO:0014824]; B cell receptor signaling pathway [GO:0050853]; female pregnancy [GO:0007565]; long-term synaptic depression [GO:0060292]; negative regulation of apoptotic process [GO:0043066]; negative regulation of bone resorption [GO:0045779]; negative regulation of neuron projection development [GO:0010977]; negative regulation of transcription, DNA-templated [GO:0045892]; positive regulation of B cell proliferation [GO:0030890]; positive regulation of cell growth [GO:0030307]; positive regulation of cell population proliferation [GO:0008284]; positive regulation of cytosolic calcium ion concentration [GO:0007204]; positive regulation of insulin secretion [GO:0032024]; positive regulation of transcription, DNA-templated [GO:0045893]; positive regulation of vasoconstriction [GO:0045907]; response to estradiol [GO:0032355]; response to hormone [GO:0009725]; response to hydroperoxide [GO:0033194]; response to hypoxia [GO:0001666]; response to interleukin-1 [GO:0070555]; response to progesterone [GO:0032570]; response to retinoic acid [GO:0032526]; response to xenobiotic stimulus [GO:0009410]
CRHBP_MOUSE	Corticotropin-releasing factor-binding protein	Crhbp	behavioral response to ethanol [GO:0048149]; cellular response to calcium ion [GO:0071277]; cellular response to cAMP [GO:0071320]; cellular response to cocaine [GO:0071314]; cellular response to estradiol stimulus [GO:0071392]; cellular response to estrogen stimulus [GO:0071391]; cellular response to gonadotropin-releasing hormone [GO:0097211]; cellular response to immobilization stress [GO:0035903]; cellular response to potassium ion [GO:0035865]; cellular response to tumor necrosis factor [GO:0071356]; cellular response to

			xenobiotic stimulus [GO:0071466]; female pregnancy [GO:0007565]; hormone metabolic process [GO:0042445]; hormone-mediated signaling pathway [GO:0009755]; inflammatory response [GO:0006954]; maternal aggressive behavior [GO:0002125]; negative regulation of corticotropin secretion [GO:0051460]; negative regulation of corticotropin-releasing hormone receptor activity [GO:1900011]; regulated exocytosis [GO:0045055]; regulation of cellular response to stress [GO:0080135]; regulation of corticotropin secretion [GO:0051459]; regulation of NMDA receptor activity [GO:2000310]; synaptic transmission, dopaminergic [GO:0001963]
CSMD3_MOUSE	CUB and sushi domain-containing protein 3	Csmd3	regulation of dendrite development [GO:0050773]
DOC2B_MOUSE	Double C2-like domain-containing protein beta	Doc2b	calcium ion-regulated exocytosis of neurotransmitter [GO:0048791]; positive regulation of calcium ion-dependent exocytosis [GO:0045956]; positive regulation of insulin secretion [GO:0032024]; positive regulation of vesicle fusion [GO:0031340]; protein localization [GO:0008104]; spontaneous neurotransmitter secretion [GO:0061669]
DOP2_MOUSE	Protein dopey-2	Dop1b	cognition [GO:0050890]; embryonic pattern specification [GO:0009880]; Golgi to endosome transport [GO:0006895]; protein transport [GO:0015031]
EPHA5_MOUSE	Ephrin type-A receptor 5	Epha5	axon guidance [GO:0007411]; cAMP-mediated signaling [GO:0019933]; cellular response to follicle-stimulating hormone stimulus [GO:0071372]; cellular response to forskolin [GO:1904322]; dendritic spine morphogenesis [GO:0060997]; ephrin receptor signaling pathway [GO:0048013]; hippocampus development [GO:0021766]; negative regulation of cell adhesion [GO:0007162]; positive regulation of CREB transcription factor activity [GO:0032793]; positive regulation of kinase activity [GO:0033674]; regulation of actin cytoskeleton organization [GO:0032956]; regulation of GTPase activity [GO:0043087]; regulation of insulin secretion involved in cellular response to glucose stimulus [GO:0061178]; transmembrane receptor protein tyrosine kinase signaling pathway [GO:0007169]
FGF13_MOUSE	Fibrinogen C domain-containing protein 1	Fgf13	cerebral cortex cell migration [GO:0021795]; establishment of neuroblast polarity [GO:0045200]; hippocampus development [GO:0021766]; inhibitory synapse assembly [GO:1904862]; learning [GO:0007612]; MAPK cascade [GO:0000165]; memory [GO:0007613]; microtubule polymerization [GO:0046785]; negative regulation of collateral sprouting [GO:0048671]; negative regulation of microtubule depolymerization [GO:0007026]; neuron migration [GO:0001764]; protein localization to plasma membrane [GO:0072659]; regulation of cardiac muscle cell action potential involved in regulation of contraction [GO:0098909]; regulation of voltage-gated sodium channel activity [GO:1905150]; sodium ion transport [GO:0006814]
FBCD1_MOUSE	Fibroblast growth factor 13	Fibcd1	cell adhesion [GO:0007155]
FLRT2_MOUSE	Leucine-rich repeat transmembrane protein FLRT2	Flrt2	axon guidance [GO:0007411]; basement membrane organization [GO:0071711]; cell adhesion involved in heart morphogenesis [GO:0061343]; fibroblast growth factor receptor signaling pathway [GO:0008543]; heart morphogenesis [GO:0003007]; positive regulation of synapse assembly [GO:0051965]; regulation of neuron migration [GO:2001222]
FUT8_MOUSE	Alpha-	Fut8	cell migration [GO:0016477]; GDP-L-fucose metabolic process [GO:0046368]; integrin-mediated signaling pathway [GO:0007229]; N-glycan fucosylation [GO:0036071]; N-glycan processing [GO:0006491]; protein glycosylation in Golgi [GO:0033578]; protein N-linked glycosylation [GO:0006487]; protein N-linked glycosylation via asparagine [GO:0018279]; receptor metabolic process [GO:0043112]; regulation of cellular response to oxidative stress [GO:1900407]; regulation of gene

			expression [GO:0010468]; respiratory gaseous exchange by respiratory system [GO:0007585]; transforming growth factor beta receptor signaling pathway [GO:0007179]
KIRR3_MOUSE	Transcription factor 4	Kirrel3	cell-cell adhesion [GO:0098609]; glomerulus morphogenesis [GO:0072102]; hemopoiesis [GO:0030097]; hippocampus development [GO:0021766]; homophilic cell adhesion via plasma membrane adhesion molecules [GO:0007156]; inter-male aggressive behavior [GO:0002121]; neuron migration [GO:0001764]; neuron projection morphogenesis [GO:0048812]; principal sensory nucleus of trigeminal nerve development [GO:0021740]; synapse assembly [GO:0007416]
LEG3_MOUSE	Kin of IRRE-like protein 3	Lgals3	antimicrobial humoral immune response mediated by antimicrobial peptide [GO:0061844]; cell differentiation [GO:0030154]; eosinophil chemotaxis [GO:0048245]; extracellular matrix organization [GO:0030198]; innate immune response [GO:0045087]; killing of cells of another organism [GO:0031640]; macrophage chemotaxis [GO:0048246]; monocyte chemotaxis [GO:0002548]; mononuclear cell migration [GO:0071674]; mRNA processing [GO:0006397]; negative regulation of apoptotic process [GO:0043066]; negative regulation of cell proliferation in bone marrow [GO:1903769]; negative regulation of endocytosis [GO:0045806]; negative regulation of extrinsic apoptotic signaling pathway [GO:2001237]; negative regulation of immunological synapse formation [GO:2000521]; negative regulation of T cell activation via T cell receptor contact with antigen bound to MHC molecule on antigen presenting cell [GO:2001189]; negative regulation of T cell receptor signaling pathway [GO:0050860]; neutrophil chemotaxis [GO:0030593]; positive chemotaxis [GO:0050918]; positive regulation of angiogenesis [GO:0045766]; positive regulation of calcium ion import [GO:0090280]; positive regulation of cell population proliferation [GO:0008284]; positive regulation of mononuclear cell migration [GO:0071677]; positive regulation of protein localization to plasma membrane [GO:1903078]; positive regulation of protein-containing complex assembly [GO:0031334]; positive regulation of serotonin secretion [GO:0014064]; regulation of extrinsic apoptotic signaling pathway via death domain receptors [GO:1902041]; regulation of T cell apoptotic process [GO:0070232]; regulation of T cell proliferation [GO:0042129]; RNA splicing [GO:0008380]; skeletal system development [GO:0001501]
LRFN2_MOUSE	Ribosomal protein S6 kinase beta-1	Lrfn2	modulation of chemical synaptic transmission [GO:0050804]; regulation of postsynapse organization [GO:0099175]
MDGA1_MOUSE	Galectin-3	Mdga1	cerebral cortex radially oriented cell migration [GO:0021799]; negative regulation of synapse assembly [GO:0051964]; nervous system development [GO:0007399]; neuron migration [GO:0001764]; regulation of presynapse assembly [GO:1905606]; regulation of synaptic membrane adhesion [GO:0099179]
MK_MOUSE	Leucine-rich repeat and fibronectin type-III domain-containing protein 2	Mdk	adrenal gland development [GO:0030325]; behavioral fear response [GO:0001662]; cell migration [GO:0016477]; cerebellar granular layer development [GO:0021681]; cerebral cortex development [GO:0021987]; cytoskeleton organization [GO:0007010]; defecation [GO:0030421]; dentate gyrus development [GO:0021542]; estrous cycle [GO:0044849]; glial cell projection elongation [GO:0106091]; hippocampus development [GO:0021766]; leukocyte chemotaxis involved in inflammatory response [GO:0002232]; negative regulation of canonical Wnt signaling pathway [GO:0090090]; negative regulation of cardiac muscle cell apoptotic process [GO:0010667]; negative regulation of cell adhesion [GO:0007162]; negative regulation of epithelial cell apoptotic process [GO:1904036]; negative regulation of inflammatory response to wounding [GO:0106015]; negative regulation of neuron apoptotic process [GO:0043524]; negative regulation of neuron death [GO:1901215]; negative regulation of ossification [GO:0030279]; negative regulation of regulatory T cell differentiation [GO:0045590]; oogenesis [GO:0048477]; positive

			regulation of artery morphogenesis [GO:1905653]; positive regulation of blood vessel branching [GO:1905555]; positive regulation of cartilage development [GO:0061036]; positive regulation of cell adhesion [GO:0045785]; positive regulation of cell division [GO:0051781]; positive regulation of cell migration [GO:0030335]; positive regulation of epithelial to mesenchymal transition [GO:0010718]; positive regulation of hepatocyte proliferation [GO:2000347]; positive regulation of inflammatory response [GO:0050729]; positive regulation of inflammatory response to wounding [GO:0106016]; positive regulation of interleukin-12 production [GO:0032735]; positive regulation of keratinocyte proliferation [GO:0010838]; positive regulation of leukocyte adhesion to vascular endothelial cell [GO:1904996]; positive regulation of leukocyte cell-cell adhesion [GO:1903039]; positive regulation of leukocyte chemotaxis [GO:0002690]; positive regulation of macrophage chemotaxis [GO:0010759]; positive regulation of neural precursor cell proliferation [GO:2000179]; positive regulation of neuron migration [GO:2001224]; positive regulation of neuron projection development [GO:0010976]; positive regulation of neutrophil chemotaxis [GO:0090023]; positive regulation of neutrophil extravasation [GO:2000391]; positive regulation of oligodendrocyte differentiation [GO:0048714]; positive regulation of smooth muscle cell chemotaxis [GO:0071673]; positive regulation of substrate adhesion-dependent cell spreading [GO:1900026]; positive regulation of T cell differentiation [GO:0045582]; positive regulation of transcription, DNA-templated [GO:0045893]; positive regulation of vascular endothelial cell proliferation [GO:1905564]; regulation of actin cytoskeleton reorganization [GO:2000249]; regulation of behavior [GO:0050795]; regulation of bone remodeling [GO:0046850]; regulation of chondrocyte differentiation [GO:0032330]; response to auditory stimulus [GO:0010996]; response to glucocorticoid [GO:0051384]; response to wounding [GO:0009611]; response to xenobiotic stimulus [GO:0009410]; short-term memory [GO:0007614]; T cell activation involved in immune response [GO:0002286]; tissue regeneration [GO:0042246]
MT3_MOUSE	MAM domain-containing glycosylphosphatidylinositol anchor protein 1	Mt3	activation of protein kinase B activity [GO:0032148]; astrocyte development [GO:0014002]; cadmium ion homeostasis [GO:0055073]; cellular lipid catabolic process [GO:0044242]; cellular metal ion homeostasis [GO:0006875]; cellular response to cadmium ion [GO:0071276]; cellular response to copper ion [GO:0071280]; cellular response to nitric oxide [GO:0071732]; cellular response to oxidative stress [GO:0034599]; cellular response to zinc ion [GO:0071294]; cellular zinc ion homeostasis [GO:0006882]; cholesterol catabolic process [GO:0006707]; detoxification of copper ion [GO:0010273]; energy reserve metabolic process [GO:0006112]; ERK1 and ERK2 cascade [GO:0070371]; histone modification [GO:0016570]; leptin-mediated signaling pathway [GO:0033210]; negative regulation of apoptotic process [GO:0043066]; negative regulation of autophagy [GO:0010507]; negative regulation of axon extension [GO:0030517]; negative regulation of cell growth [GO:0030308]; negative regulation of cysteine-type endopeptidase activity [GO:2000117]; negative regulation of cysteine-type endopeptidase activity involved in apoptotic process [GO:0043154]; negative regulation of hydrogen peroxide catabolic process [GO:2000296]; negative regulation of necrotic cell death [GO:0060547]; negative regulation of neurogenesis [GO:0050768]; negative regulation of neuron apoptotic process [GO:0043524]; negative regulation of neuron death [GO:1901215]; negative regulation of oxidoreductase activity [GO:0051354]; negative regulation of reactive oxygen species metabolic process [GO:2000378]; negative regulation of transcription, DNA-templated [GO:0045892]; positive regulation of catalytic activity [GO:0043085]; positive regulation of cell death [GO:0010942]; positive regulation of ERK1 and ERK2 cascade [GO:0070374]; positive regulation of gene expression [GO:0010628]; positive

			regulation of lysosomal membrane permeability [GO:0097214]; positive regulation of necrotic cell death [GO:0010940]; positive regulation of oxygen metabolic process [GO:2000376]; positive regulation of protein phosphorylation [GO:0001934]; positive regulation of transcription from RNA polymerase II promoter in response to oxidative stress [GO:0036091]; positive regulation of transcription, DNA-templated [GO:0045893]; positive regulation of vascular endothelial growth factor receptor signaling pathway [GO:0030949]; protein kinase B signaling [GO:0043491]; protein stabilization [GO:0050821]; regulation of protein glycosylation [GO:0060049]; regulation of response to food [GO:0032095]; removal of superoxide radicals [GO:0019430]; response to hypoxia [GO:0001666]; response to oxidative stress [GO:0006979]; zinc ion homeostasis [GO:0055069]; zinc ion transport [GO:0006829]
MYD88_MOUSE	Midkine	Myd88	3'-UTR-mediated mRNA stabilization [GO:0070935]; apoptotic process [GO:0006915]; cell surface receptor signaling pathway [GO:0007166]; cellular response to lipopolysaccharide [GO:0071222]; cellular response to mechanical stimulus [GO:0071260]; cellular response to oxidised low-density lipoprotein particle stimulus [GO:0140052]; cytokine-mediated signaling pathway [GO:0019221]; defense response to bacterium [GO:0042742]; defense response to Gram-positive bacterium [GO:0050830]; defense response to protozoan [GO:0042832]; defense response to virus [GO:0051607]; establishment of endothelial intestinal barrier [GO:0090557]; immune response [GO:0006955]; immunoglobulin mediated immune response [GO:0016064]; induced systemic resistance [GO:0009682]; inflammatory response [GO:0006954]; innate immune response [GO:0045087]; interleukin-1-mediated signaling pathway [GO:0070498]; JNK cascade [GO:0007254]; leukocyte activation involved in inflammatory response [GO:0002269]; lipopolysaccharide-mediated signaling pathway [GO:0031663]; MyD88-dependent toll-like receptor signaling pathway [GO:0002755]; neutrophil activation involved in immune response [GO:0002283]; neutrophil-mediated killing of bacterium [GO:0070944]; phagocytosis [GO:0006909]; positive regulation of chemokine production [GO:0032722]; positive regulation of cytokine production involved in inflammatory response [GO:1900017]; positive regulation of gene expression [GO:0010628]; positive regulation of I-kappaB kinase/NF-kappaB signaling [GO:0043123]; positive regulation of interleukin-1 beta production [GO:0032731]; positive regulation of interleukin-17 production [GO:0032740]; positive regulation of interleukin-23 production [GO:0032747]; positive regulation of interleukin-6 production [GO:0032755]; positive regulation of interleukin-8 production [GO:0032757]; positive regulation of JNK cascade [GO:0046330]; positive regulation of lymphocyte proliferation [GO:0050671]; positive regulation of NF-kappaB transcription factor activity [GO:0051092]; positive regulation of NLRP3 inflammasome complex assembly [GO:1900227]; positive regulation of smooth muscle cell proliferation [GO:0048661]; positive regulation of tumor necrosis factor production [GO:0032760]; positive regulation of type I interferon production [GO:0032481]; regulation of cell population proliferation [GO:0042127]; regulation of chemokine (C-X-C motif) ligand 1 production [GO:2000338]; regulation of chemokine (C-X-C motif) ligand 2 production [GO:2000341]; regulation of gene expression [GO:0010468]; regulation of inflammatory response [GO:0050727]; regulation of interleukin-6 production [GO:0032675]; regulation of neutrophil migration [GO:1902622]; regulation of tumor necrosis factor production [GO:0032680]; response to interleukin-1 [GO:0070555]; response to lipopolysaccharide [GO:0032496]; response to molecule of fungal origin [GO:0002238]; response to peptidoglycan [GO:0032494]; response to virus [GO:0009615]; Toll signaling pathway [GO:0008063]; toll-like receptor 8 signaling pathway [GO:0034158]; transmembrane receptor protein serine/threonine kinase signaling pathway

			[GO:0007178]; type I interferon signaling pathway [GO:0060337]
NTNG2_MOUSE	Metallothionein-3	Ntng2	animal organ morphogenesis [GO:0009887]; axonogenesis [GO:0007409]; basement membrane assembly [GO:0070831]; cell migration [GO:0016477]; modulation of chemical synaptic transmission [GO:0050804]; postsynaptic specialization assembly [GO:0098698]; regulation of neuron migration [GO:2001222]; regulation of neuron projection arborization [GO:0150011]; regulation of neuron projection development [GO:0010975]; regulation of presynapse assembly [GO:1905606]; substrate adhesion-dependent cell spreading [GO:0034446]; synaptic membrane adhesion [GO:0099560]; tissue development [GO:0009888]
PK3CG_MOUSE	Myeloid differentiation primary response protein MyD88	Pik3cg	angiogenesis [GO:0001525]; cell migration [GO:0016477]; cellular response to cAMP [GO:0071320]; chemotaxis [GO:0006935]; endocytosis [GO:0006897]; G protein-coupled receptor signaling pathway [GO:0007186]; hepatocyte apoptotic process [GO:0097284]; immune system process [GO:0002376]; inflammatory response [GO:0006954]; negative regulation of fibroblast apoptotic process [GO:2000270]; negative regulation of triglyceride catabolic process [GO:0010897]; phosphatidylinositol 3-kinase signaling [GO:0014065]; phosphatidylinositol phosphate biosynthetic process [GO:0046854]; phosphatidylinositol-3-phosphate biosynthetic process [GO:0036092]; phosphatidylinositol-mediated signaling [GO:0048015]; phosphorylation [GO:0016310]; positive regulation of acute inflammatory response [GO:0002675]; positive regulation of catalytic activity [GO:0043085]; positive regulation of cytosolic calcium ion concentration [GO:0007204]; positive regulation of endothelial cell migration [GO:0010595]; positive regulation of MAP kinase activity [GO:0043406]; positive regulation of protein kinase B signaling [GO:0051897]; positive regulation of Rac protein signal transduction [GO:0035022]; protein phosphorylation [GO:0006468]; regulation of calcium ion transmembrane transport [GO:1903169]; regulation of protein phosphorylation [GO:0001932]; secretory granule localization [GO:0032252]; sphingosine-1-phosphate receptor signaling pathway [GO:0003376]
KS6B1_MOUSE	Netrin-G2	Rps6kb1	aging [GO:0007568]; apoptotic process [GO:0006915]; behavioral fear response [GO:0001662]; cell migration [GO:0016477]; cellular response to dexamethasone stimulus [GO:0071549]; cellular response to growth factor stimulus [GO:0071363]; cellular response to insulin stimulus [GO:0032869]; cellular response to interferon-gamma [GO:0071346]; cellular response to organic cyclic compound [GO:0071407]; G1/S transition of mitotic cell cycle [GO:0000082]; germ cell development [GO:0007281]; long-chain fatty acid import into cell [GO:0044539]; long-term memory [GO:0007616]; negative regulation of apoptotic process [GO:0043066]; negative regulation of extrinsic apoptotic signaling pathway [GO:2001237]; negative regulation of insulin receptor signaling pathway [GO:0046627]; peptidyl-serine phosphorylation [GO:0018105]; positive regulation of mitotic cell cycle [GO:0045931]; positive regulation of skeletal muscle tissue growth [GO:0048633]; positive regulation of smooth muscle cell migration [GO:0014911]; positive regulation of smooth muscle cell proliferation [GO:0048661]; positive regulation of translation [GO:0045727]; positive regulation of translational initiation [GO:0045948]; protein kinase B signaling [GO:0043491]; protein phosphorylation [GO:0006468]; regulation of glucose import [GO:0046324]; response to electrical stimulus involved in regulation of muscle adaptation [GO:0014878]; response to ethanol [GO:0045471]; response to glucagon [GO:0033762]; response to glucose [GO:0009749]; response to heat [GO:0009408]; response to insulin [GO:0032868]; response to leucine [GO:0043201]; response to lipopolysaccharide [GO:0032496]; response to mechanical stimulus [GO:0009612]; response to nutrient [GO:0007584]; response to nutrient levels [GO:0031667]; response to

			testosterone [GO:0033574]; response to toxic substance [GO:0009636]; response to tumor necrosis factor [GO:0034612]; response to wounding [GO:0009611]; response to xenobiotic stimulus [GO:0009410]; skeletal muscle atrophy [GO:0014732]; skeletal muscle contraction [GO:0003009]; TOR signaling [GO:0031929]
RTN4R_MOUSE	Phosphatidylinositol 4	Rtn4r	axon guidance [GO:0007411]; axonogenesis [GO:0007409]; cell surface receptor signaling pathway [GO:0007166]; corpus callosum development [GO:0022038]; negative chemotaxis [GO:0050919]; negative regulation of axon extension [GO:0030517]; negative regulation of axon regeneration [GO:0048681]; negative regulation of neuron projection development [GO:0010977]; neuronal signal transduction [GO:0023041]; positive regulation of GTPase activity [GO:0043547]; positive regulation of Rho protein signal transduction [GO:0035025]
SORC3_MOUSE	Reticulon-4 receptor	Sorcs3	learning [GO:0007612]; memory [GO:0007613]; regulation of long-term synaptic depression [GO:1900452]
TAFA5_MOUSE	VPS10 domain-containing receptor SorCS3	Tafa5	G protein-coupled receptor signaling pathway [GO:0007186]; negative regulation of vascular associated smooth muscle cell migration [GO:1904753]; negative regulation of vascular associated smooth muscle cell proliferation [GO:1904706]; negative regulation of vascular wound healing [GO:0061044]
ITF2_MOUSE	Chemokine-like protein TAFA-5	Tcf4	cell differentiation [GO:0030154]; endothelial cell activation [GO:0042118]; negative regulation of angiogenesis [GO:0016525]; negative regulation of gene expression [GO:0010629]; negative regulation of transcription by RNA polymerase II [GO:0000122]; nervous system development [GO:0007399]; positive regulation of neuron differentiation [GO:0045666]; positive regulation of transcription by RNA polymerase II [GO:0045944]; positive regulation of transcription, DNA-templated [GO:0045893]; protein-DNA complex assembly [GO:0065004]; regulation of transcription by RNA polymerase II [GO:0006357]; regulation of transcription, DNA-templated [GO:0006355]; regulation of vascular endothelial growth factor signaling pathway [GO:1900746]
TRPC4_MOUSE	Short transient receptor potential channel 4	Trpc4	calcium ion import [GO:0070509]; calcium ion transmembrane transport [GO:0070588]; calcium ion transport [GO:0006816]; gamma-aminobutyric acid secretion [GO:0014051]; manganese ion transport [GO:0006828]; oligodendrocyte differentiation [GO:0048709]; regulation of action potential firing rate [GO:0099605]; regulation of calcium ion transport [GO:0051924]; regulation of cytosolic calcium ion concentration [GO:0051480]
ZEB2_MOUSE	Zinc finger E-box-binding homeobox 2	Zeb2	anatomical structure development [GO:0048856]; cell proliferation in forebrain [GO:0021846]; central nervous system development [GO:0007417]; collateral sprouting [GO:0048668]; corpus callosum morphogenesis [GO:0021540]; corticospinal tract morphogenesis [GO:0021957]; developmental pigmentation [GO:0048066]; embryonic morphogenesis [GO:0048598]; hippocampus development [GO:0021766]; mammillary axonal complex development [GO:0061373]; melanocyte migration [GO:0097324]; negative regulation of transcription by RNA polymerase II [GO:0000122]; neural crest cell migration [GO:0001755]; neural tube closure [GO:0001843]; positive regulation of axonogenesis [GO:0050772]; positive regulation of canonical Wnt signaling pathway [GO:0090263]; positive regulation of JUN kinase activity [GO:0043507]; positive regulation of lens fiber cell differentiation [GO:1902748]; positive regulation of melanin biosynthetic process [GO:0048023]; positive regulation of melanocyte differentiation [GO:0045636]; positive regulation of transcription by RNA polymerase II [GO:0045944]; positive regulation of transforming growth factor beta receptor signaling pathway [GO:0030511]; positive regulation of Wnt signaling pathway [GO:0030177]; regulation of melanosome organization [GO:1903056]; regulation of transcription by RNA polymerase II [GO:0006357]; somitogenesis [GO:0001756]

Reference list due to particular importance for the area specific functionality belonging to 27 proteins from Table 1

Pubmed search keywords (hippocampus + "gene name"); publication hits were filtered based on the following exclusion criteria: (i) data matching another subregion of the hippocampus, such as the dentate gyrus, and (ii) data analyzing adult neurogenesis (since adult neurogenesis does not occur in the CA1 region).

CD38

1: Nelissen TP, Bamford RA, Tochitani S, Akkus K, Kudzinskas A, Yokoi K, Okamoto H, Yamamoto Y, Burbach JPH, Matsuzaki H, Oguro-Ando A. CD38 is Required for **Dendritic Organization** in Visual Cortex and Hippocampus. Neuroscience. 2018 Feb 21;372:114-125. doi: 10.1016/j.neuroscience.2017.12.050. Epub 2018 Jan 3. PMID: 29306053.

2: Kim S, Kim T, Lee HR, Jang EH, Ryu HH, Kang M, Rah SY, Yoo J, Lee B, Kim JI, Lim CS, Kim SJ, Kim UH, Lee YS, Kaang BK. **Dendritic Organization** and memory in CD38 null mutant mice. Mol Brain. 2016 Feb 9;9:16. doi: 10.1186/s13041-016-0195-5. PMID: 26856703; PMCID: PMC4746819.

3: Khodaverdian S, Dashtban-Moghadam E, Dabirmanesh B, Mirnajafi-Zadeh J, Taleb M, Khajeh K, Fathollahi Y. CD38 and MGluR1 as possible signaling molecules involved in **epileptogenesis**: A potential role for NAD+ homeostasis. Brain Res. 2021 Aug 15;1765:147509. doi: 10.1016/j.brainres.2021.147509. Epub 2021 Apr 28. PMID: 33930374.

4: Reyes-Harde M, Empson R, Potter BV, Galione A, Stanton PK. Evidence of a role for cyclic ADP-ribose in **long-term synaptic depression** in hippocampus. Proc Natl Acad Sci U S A. 1999 Mar 30;96(7):4061-6. doi: 10.1073/pnas.96.7.4061. PMID: 10097163; PMCID: PMC22420.

Fut8:

1: Gu W, Fukuda T, Isaji T, Hang Q, Lee HH, Sakai S, Morise J, Mitoma J, Higashi H, Taniguchi N, Yawo H, Oka S, Gu J. Loss of α1,6-Fucosyltransferase **Decreases Hippocampal Long Term Potentiation**: IMPLICATIONS FOR CORE FUCOSYLATION IN THE REGULATION OF AMPA RECEPTOR HETEROMERIZATION AND CELLULAR SIGNALING. J Biol Chem. 2015 Jul 10;290(28):17566-75. doi: 10.1074/jbc.M114.579938. Epub 2015 May 15. PMID: 25979332; PMCID: PMC4498090.

APOH:

1: Zou S, Zhang J; for Alzheimer's Disease Neuroimaging Initiative; Chen W. Subtypes Based on Six Apolipoproteins in Non-Demented Elderly Are Associated **with Cognitive Decline and** Subsequent Tau Accumulation in erebrospinal Fluid. J Alzheimers Dis. 2019;72(2):413-423. doi: 10.3233/JAD-190314. PMID: 31594221.

TAFA5:

1: Huang S, Zheng C, Xie G, Song Z, Wang P, Bai Y, Chen D, Zhang Y, Lv P, Liang W, She S, Li Q, Liu Z, Wang Y, Xing GG, Wang Y. FAM19A5/TAFA5, a novel neurokine, plays a crucial r**ole in depressive-like and spatial memory-related behaviors in mice**. Mol Psychiatry. 2021 Jun;26(6):2363-2379. doi: 10.1038/s41380-020-0720-x. Epub 2020 Apr 21. PMID: 32317715.

CRHBP:

1: Comasco E, Schijven D, de Maeyer H, Vrettou M, Nylander I, Sundström-Poromaa I, Olivier JDA. Constitutive Serotonin Transporter Reduction Resembles Maternal **Separation with Regard to Stress-Related** Gene Expression. ACS Chem Neurosci. 2019 Jul 17;10(7):3132-3142. doi: 10.1021/acschemneuro.8b00595. Epub 2019 Jan 23. PMID: 30614673.

2: Lombardo KA, Herringa RJ, Balachandran JS, Hsu DT, Bakshi VP, Roseboom PH, Kalin NH. Effects of acute and repeated **restraint stress** on corticotropin-releasing hormone binding protein mRNA in rat amygdala and dorsal hippocampus. Neurosci Lett. 2001 Apr 20;302(2-3):81-4. doi: 10.1016/s0304-3940(01)01680-9. PMID: 11290392.

3: Li WX, Dai SX, Liu JQ, Wang Q, Li GH, Huang JF. Integrated Analysis of **Alzheimer's Disease and Schizophrenia** Dataset Revealed Different Expression Pattern in Learning and Memory. J Alzheimers Dis. 2016;51(2):417-25. doi: 10.3233/JAD-150807. PMID: 26890750.

4: Ketchesin KD, Huang NS, Seasholtz AF. Cell Type-Specific Expression of Corticotropin-Releasing Hormone-Binding Protein in GABAergic Interneurons in the Prefrontal Cortex. Front Neuroanat. 2017 Oct 10;11:90. doi: 10.3389/fnana.2017.00090. PMID: 29066956; PMCID: PMC5641307.

5: Lisowski P, Juszczak GR, Goscik J, Stankiewicz AM, Wieczorek M, Zwierzchowski L, Swiergiel AH. **Stress susceptibility**-specific phenotype associated with different hippocampal transcriptomic responses to chronic tricyclic antidepressant treatment in mice. BMC Neurosci. 2013 Nov 13;14:144. doi: 10.1186/1471-2202-14-144. PMID: 24225037; PMCID: PMC3831054.

CSMD3:
1: Mizukami T, Kohno T, Hattori M. CUB and Sushi multiple domains 3 regulates **dendrite development**. Neurosci Res. 2016 Sep;110:11-7. doi: 10.1016/j.neures.2016.03.003. Epub 2016 Mar 28. PMID: 27033969.

DOC2B:
1: Groffen AJ, Friedrich R, Brian EC, Ashery U, Verhage M. DOC2A and DOC2B are sensors for **neuronal activity** with unique calcium-dependent and kinetic properties. J Neurochem. 2006 May;97(3):818-33. doi: 10.1111/j.1471-4159.2006.03755.x. Epub 2006 Mar 3. PMID: 16515538.
2: Bourgeois-Jaarsma Q, Verhage M, Groffen AJ. Doc2b Ca2+ binding site **mutants enhance synaptic release at rest at the expense of sustained synaptic strength**. Sci Rep. 2019 Oct 8;9(1):14408. doi: 10.1038/s41598-019-50684-1. PMID: 31594980; PMCID: PMC6783474.
3: Groffen AJ, Martens S, Díez Arazola R, Cornelisse LN, Lozovaya N, de Jong AP, Goriounova NA, Habets RL, Takai Y, Borst JG, Brose N, McMahon HT, Verhage M. Doc2b is a high-affinity Ca2+ **sensor for spontaneous neurotransmitter release**. Science. 2010 Mar 26;327(5973):1614-8. doi: 10.1126/science.1183765. Epub 2010 Feb 11. Erratum in: Science. 2010 May 7;328(5979):690. PMID: 20150444; PMCID: PMC2846320.
4: Xue R, Gaffaney JD, Chapman ER. Structural elements that underlie Doc2β function during asynchronous **synaptic transmission**. Proc Natl Acad Sci U S A. 2015 Aug 4;112(31):E4316-25. doi: 10.1073/pnas.1502288112. Epub 2015 Jul 20. PMID: 26195798; PMCID: PMC4534243.
5: Yao J, Gaffaney JD, Kwon SE, Chapman ER. Doc2 is a Ca2+ sensor required for **asynchronous neurotransmitter release**. Cell. 2011 Oct 28;147(3):666-77. doi: 10.1016/j.cell.2011.09.046. PMID: 22036572; PMCID: PMC3220409.
6: Bourgeois-Jaarsma Q, Miaja Hernandez P, Groffen AJ. Ca²⁺ sensor proteins in spontaneous release and **synaptic plasticity**: Limited contribution of Doc2c, rabphilin-3a and synaptotagmin 7 in hippocampal glutamatergic neurons. Mol Cell Neurosci. 2021 Apr;112:103613. doi: 10.1016/j.mcn.2021.103613. Epub 2021 Mar 19. PMID: 33753311.

EphA5:
1: Liu TT, Xiao B, Li SY, Li GL, Lu XQ, Liu WP. [Changes in the expression of EphA5/ephrinA5 in the CA3 region of the hippocampus in rats with **epilepsy** and their role in the pathogenesis of temporal lobe epilepsy]. Zhongguo Dang Dai Er Ke Za Zhi. 2017 Dec;19(12):1272-1277. Chinese. doi:10.7499/j.issn.1008-8830.2017.12.010. PMID: 29237529; PMCID: PMC7389806.
2: Numachi Y, Yoshida S, Yamashita M, Fujiyama K, Toda S, Matsuoka H, Kajii Y, Nishikawa T. Altered EphA5 mRNA expression in rat brain with a single **methamphetamine** treatment. Neurosci Lett. 2007 Sep 7;424(2):116-21. doi: 10.1016/j.neulet.2007.07.025. Epub 2007 Aug 1. PMID: 17714871.
3: Gao WQ, Shinsky N, Armanini MP, Moran P, Zheng JL, Mendoza-Ramirez JL, Phillips HS, Winslow JW, Caras IW. Regulation of hippocampal **synaptic plasticity** by the tyrosine kinase receptor, REK7/EphA5, and its ligand, AL-1/Ephrin-A5. Mol Cell Neurosci. 1998 Aug;11(5-6):247-59. doi: 10.1006/mcne.1998.0696. PMID:9698392.
4: Akaneya Y, Sohya K, Kitamura A, Kimura F, Washburn C, Zhou R, Ninan I, Tsumoto T, Ziff EB. Ephrin-A5 and EphA5 interaction induces **synaptogenesis** during early hippocampal development. PLoS One. 2010 Aug 31;5(8):e12486. doi: 10.1371/journal.pone.0012486. PMID: 20824214; PMCID: PMC2930854.
5: Martínez A, Otal R, Sieber BA, Ibáñez C, Soriano E. Disruption of ephrin-A/EphA binding alters **synaptogenesis** and neural connectivity in the hippocampus. Neuroscience. 2005;135(2):451-61. doi: 10.1016/j.neuroscience.2005.06.052. PMID: 16112477.

FIBCD1
1: Fell CW, Hagelkruys A, Cicvaric A, Horrer M, Liu L, Li JSS, Stadlmann J, Polyansky AA, Mereiter S, Tejada MA, Kokotović T, Achuta VS, Scaramuzza A, Twyman KA, Morrow MM, Juusola J, Yan H, Wang J, Burmeister M, Choudhury B, Andersen TL, Wirnsberger G, Holmskov U, Perrimon N, Žagrović B, Monje FJ, Moeller JB, Penninger JM, Nagy V. FIBCD1 is an endocytic GAG receptor associated with a novel **neurodevelopmental disorder.** EMBO Mol Med. 2022 Sep 7;14(9):e15829. doi: 10.15252/emmm.202215829. Epub 2022 Aug 2. PMID: 35916241; PMCID: PMC9449597.

Lgals3 (Galectin-3)
1: Chen YC, Ma YL, Lin CH, Cheng SJ, Hsu WL, Lee EH. Galectin-3 Negatively Regulates **Hippocampus-Dependent Memory Formation** through Inhibition of Integrin Signaling and Galectin-3 Phosphorylation. Front Mol Neurosci. 2017 Jul 11;10:217. doi: 10.3389/fnmol.2017.00217. PMID: 28744198; PMCID: PMC5504160.
2: Stajic D, Selakovic D, Jovicic N, Joksimovic J, Arsenijevic N, Lukic ML, Rosic G. The role of galectin-3 in modulation of anxiety state level in mice. Brain Behav Immun. 2019 May;78:177-187. doi: 10.1016/j.bbi.2019.01.019. Epub 2019 Jan 23. PMID: 30682502.

FGF13
1: Puranam RS, He XP, Yao L, Le T, Jang W, Rehder CW, Lewis DV, McNamara JO. Disruption of Fgf13 **causes synaptic excitatory-inhibitory imbalance** and genetic epilepsy and febrile seizures plus. J Neurosci. 2015 Jun 10;35(23):8866-81. doi: 10.1523/JNEUROSCI.3470-14.2015. PMID: 26063919; PMCID: PMC4461691.
2: Shen KF, Yue J, Wu ZF, Wu KF, Zhu G, Yang XL, Wang ZK, Wang J, Liu SY, Yang H, Zhang CQ. Fibroblast growth factor 13 is involved in the **pathogenesis of temporal lobe epilepsy**. Cereb Cortex. 2022 Nov 21;32(23):5259-5272. doi: 10.1093/cercor/bhac012. PMID: 35195262.
3: Wu QF, Yang L, Li S, Wang Q, Yuan XB, Gao X, Bao L, Zhang X. Fibroblast growth factor 13 is a microtubule-stabilizing protein regulating **neuronal polarization and migration**. Cell. 2012 Jun 22;149(7):1549-64. doi: 10.1016/j.cell.2012.04.046. PMID: 22726441.
4: Li RM, Xiao L, Zhang T, Ren D, Zhu H. Overexpression of fibroblast growth factor 13 ameliorates **amyloid-β-induced neuronal damage**. Neural Regen Res. 2023 Jun;18(6):1347-1353. doi: 10.4103/1673-5374.357902. PMID: 36453422; PMCID: PMC9838149.

Kirrel3
1: Martin EA, Muralidhar S, Wang Z, Cervantes DC, Basu R, Taylor MR, Hunter J, Cutforth T, Wilke SA, Ghosh A, Williams ME. **The intellectual disability gene Kirrel3** regulates target-specific mossy fiber synapse development in the hippocampus. Elife. 2015 Nov 17;4:e09395. doi: 10.7554/eLife.09395. Erratum in: Elife. 2016;5. pii: e18706. doi: 10.7554/eLife.18706. PMID: 26575286; PMCID: PMC4642954.

Lrfn2
1: Morimura N, Yasuda H, Yamaguchi K, Katayama KI, Hatayama M, Tomioka NH, Odagawa M, Kamiya A, Iwayama Y, Maekawa M, Nakamura K, Matsuzaki H, Tsujii M, Yamada K, Yoshikawa T, Aruga J. **Autism-like behaviours** and enhanced memory formation and synaptic plasticity in Lrfn2/SALM1-deficient mice. Nat Commun.2017 Jun 12;8:15800. doi: 10.1038/ncomms15800. PMID: 28604739; PMCID:PMC5472790.
2: McMillan KJ, Banks PJ, Hellel FL, Carmichael RE, Clairfeuille T, Evans AJ, Heesom KJ, Lewis P, Collins BM, Bashir ZI, Henley JM, Wilkinson KA, Cullen PJ. Sorting nexin-27 regulates AMPA receptor trafficking through the synaptic adhesion protein LRFN2. Elife. 2021 Jul 12;10:e59432. doi: 10.7554/eLife.59432. PMID: 34251337; PMCID: PMC8296521.

FLRT2
1: Cicvaric A, Yang J, Bulat T, Zambon A, Dominguez-Rodriguez M, Kühn R, Sadowicz MG, Siwert A, Egea J, Pollak DD, Moeslinger T, Monje FJ. Enhanced **synaptic plasticity and spatial memory** in female but not male FLRT2-haplodeficient mice. Sci Rep. 2018 Feb 27;8(1):3703. doi: 10.1038/s41598-018-22030-4. PMID: 29487336; PMCID: PMC5829229.
2: Schroeder A, Vanderlinden J, Vints K, Ribeiro LF, Vennekens KM, Gounko NV, Wierda KD, de Wit J. A Modular Organization of LRR Protein-Mediated Synaptic Adhesion Defines **Synapse Identity**. Neuron. 2018 Jul 25;99(2):329-344.e7. doi: 10.1016/j.neuron.2018.06.026. Epub 2018 Jul 5. PMID: 29983322.

MDGA1
1: Kim J, Kim S, Kim H, Hwang IW, Bae S, Karki S, Kim D, Ogelman R, Bang G, Kim JY, Kajander T, Um JW, Oh WC, Ko J. MDGA1 negatively regulates amyloid precursor protein-mediated **synapse inhibition** in the hippocampus. Proc Natl Acad Sci U S A. 2022 Jan 25;119(4):e2115326119. doi: 10.1073/pnas.2115326119. PMID: 35074912; PMCID: PMC8795569.
2: Connor SA, Ammendrup-Johnsen I, Kishimoto Y, Karimi Tari P, Cvetkovska V, Harada T, Ojima D, Yamamoto T, Wang YT, Craig AM. Loss of Synapse Repressor MDGA1 Enhances Perisomatic Inhibition, Confers Resistance to **Network Excitation**, and Impairs Cognitive Function. Cell Rep. 2017 Dec 26;21(13):3637-3645. doi: 10.1016/j.celrep.2017.11.109. PMID: 29281813.
3: Lee K, Kim Y, Lee SJ, Qiang Y, Lee D, Lee HW, Kim H, Je HS, Südhof TC, Ko J. MDGAs interact selectively with neuroligin-2 but not other neuroligins to regulate inhibitory **synapse development**. Proc Natl Acad Sci U S A. 2013 Jan 2;110(1):336-41. doi: 10.1073/pnas.1219987110. Epub 2012 Dec 17. PMID: 23248271; PMCID: PMC3538197.

Myd88
1: Nishizaki T. IL-33 Acts to Express Schaffer Collateral/CA1 LTP and **Regulate Learning and Memory by Targeting MyD88**. Neural Plast. 2017;2017:2531453. doi: 10.1155/2017/2531453. Epub 2017 Sep 24. PMID: 29147584; PMCID: PMC5632899.
2: Gao Y, Fang X, Sun H, Wang Y, Yao LJ, Li JP, Tong Y, Zhang B, Liu Y. Toll-like receptor 4-mediated myeloid differentiation factor 88-dependent **signaling pathway** is activated by cerebral ischemia-reperfusion in hippocampal CA1 region in mice. Biol Pharm Bull. 2009 Oct;32(10):1665-71. doi: 10.1248/bpb.32.1665. PMID: 19801825.
3: Schroeder P, Rivalan M, Zaqout S, Krüger C, Schüler J, Long M, Meisel A, Winter Y, Kaindl AM, Lehnardt S: **Abnormal brain structure and behavior** in MyD88-deficient mice. Brain Behav Immun. 2021 Jan;91:181-193. doi: 10.1016/j.bbi.2020.09.024. PMID: 33002631

Mt3
1: Ma F, Wang H, Chen B, Wang F, Xu H. Metallothionein 3 attenuated the apoptosis of neurons in the CA1 region of the hippocampus in the senescence-accelerated mouse/PRONE8 (SAMP8). Arq Neuropsiquiatr. 2011 Feb;69(1):105-11. doi: 10.1590/s0004-282x2011000100020. PMID: 21359432.
2: Manso Y, Carrasco J, Comes G, Meloni G, Adlard PA, Bush AI, Vašák M, Hidalgo J. Characterization of the role of metallothionein-3 in an animal model of **Alzheimer's disease**. Cell Mol Life Sci. 2012 Nov;69(21):3683-700. doi: 10.1007/s00018-012-1047-9. Epub 2012 Jun 22. PMID: 22722772.
3: Jang SW, Liu X, Pradoldej S, Tosini G, Chang Q, Iuvone PM, Ye K. N-acetylserotonin **activates TrkB receptor** in a circadian rhythm. Proc Natl Acad Sci U S A. 2010 Feb 23;107(8):3876-81. doi: 10.1073/pnas.0912531107. Epub 2010 Feb 4. PMID: 20133677; PMCID: PMC2840510.
4: Kiyohara ACP, Torres DJ, Hagiwara A, Pak J, Rueli RHLH, Shuttleworth CWR, Bellinger FP. Selenoprotein P Regulates **Synaptic Zinc and Reduces Tau Phosphorylation**. Front Nutr. 2021 Jul 1;8:683154. doi: 10.3389/fnut.2021.683154. PMID: 34277682; PMCID: PMC8280497.

Mdk
1: Meyer MA. Highly Expressed Genes within Hippocampal Sector CA1: Implications for the **Physiology of Memory**. Neurol Int. 2014 Jun 16;6(2):5388. doi: 10.4081/ni.2014.5388. PMID: 24987507; PMCID: PMC4077213.
2: Nakamura E, Kadomatsu K, Yuasa S, Muramatsu H, Mamiya T, Nabeshima T, Fan QW, Ishiguro K, Igakura T, Matsubara S, Kaname T, Horiba M, Saito H, Muramatsu T. Disruption of the midkine gene (Mdk) resulted in **altered expression of a calcium binding protein in the hippocampus of infant mice and their abnormal behaviour**. Genes Cells. 1998 Dec;3(12):811-22. doi: 10.1046/j.1365-2443.1998.00231.x. PMID: 10096022.

Ntng2
1: Pan Y, Liu G, Fang M, Shen L, Wang L, Han Y, Shen D, Wang X. Abnormal expression of netrin-G2 in **temporal lobe epilepsy neurons** in humans and a rat model. Exp Neurol. 2010 Aug;224(2):340-6. doi: 10.1016/j.expneurol.2010.04.001. Epub 2010 Apr 9. PMID: 20382146.
2: Eastwood SL, Harrison PJ. Decreased mRNA expression of netrin-G1 and netrin-G2 in the temporal lobe in **schizophrenia and bipolar disorder**. Neuropsychopharmacology. 2008 Mar;33(4):933-45. doi: 10.1038/sj.npp.1301457. Epub 2007 May 16. PMID: 17507910; PMCID: PMC2629613.
3: Xu G, Wang R, Wang Z, Lei Q, Yu Z, Liu C, Li P, Yang Z, Cheng X, Li G, Wu M. NGL-2 Is a New Partner of PAR Complex in **Axon Differentiation**. J Neurosci. 2015 May 6;35(18):7153-64. doi: 10.1523/JNEUROSCI.4726-14.2015. PMID: 25948265; PMCID: PMC6605266.

Pik3cg
1: Sim SE, Lee HR, Kim JI, Choi SL, Bakes J, Jang DJ, Lee K, Han K, Kim E, Kaang BK. Elevated RalA activity in the hippocampus of PI3Kγ knock-out mice lacking NMDAR-dependent **long-term depression**. BMB Rep. 2013 Feb;46(2):103-6. doi: 10.5483/bmbrep.2013.46.2.143. PMID: 23433113; PMCID: PMC4133848.
2: Kim JI, Lee HR, Sim SE, Baek J, Yu NK, Choi JH, Ko HG, Lee YS, Park SW, Kwak C, Ahn SJ, Choi SY, Kim H, Kim KH, Backx PH, Bradley CA, Kim E, Jang DJ, Lee K, Kim SJ, Zhuo M, Collingridge GL, Kaang BK. PI3Kγ is required for NMDA receptor-dependent **long-term depression and behavioral flexibility**. Nat Neurosci. 2011 Oct 23;14(11):1447-54. doi: 10.1038/nn.2937. PMID: 22019731.
3: Lima IV, Campos AC, Miranda AS, Vieira ÉL, Amaral-Martins F, Vago JP, Santos RP, Sousa LP, Vieira LB, Teixeira MM, Fiebich BL, Moraes MF, Teixeira AL, de Oliveira AC. PI3Kγ deficiency enhances **seizures severity** and associated outcomes in a mouse model of convulsions induced by intrahippocampal injection of pilocarpine. Exp Neurol. 2015 May;267:123-34. doi: 10.1016/j.expneurol.2015.02.021. Epub 2015 Mar 5. PMID: 25749189.
4: Passos GF, Figueiredo CP, Prediger RD, Silva KA, Siqueira JM, Duarte FS, Leal PC, Medeiros R, Calixto JB. Involvement of phosphoinositide 3-kinase gamma in the neuro-inflammatory response and **cognitive impairments induced** by beta-amyloid 1-40 peptide in mice. Brain Behav Immun. 2010 Mar;24(3):493-501. doi: 10.1016/j.bbi.2009.12.003. Epub 2009 Dec 16. PMID: 20025958.

Dop1b
1: Rachidi M, Delezoide AL, Delabar JM, Lopes C. A quantitative assessment of gene expression (QAGE) reveals differential overexpression of DOPEY2, a **candidate gene for mental retardation**, in Down syndrome brain regions. Int J Dev Neurosci. 2009 Jun;27(4):393-8. doi: 10.1016/j.ijdevneu.2009.02.001. Epub 2009 Feb 13. PMID: 19460634.

Rtn4r
1: Wills ZP, Mandel-Brehm C, Mardinly AR, McCord AE, Giger RJ, Greenberg ME. The nogo receptor family **restricts synapse number** in the developing hippocampus. Neuron. 2012 Feb 9;73(3):466-81. doi: 10.1016/j.neuron.2011.11.029. PMID: 22325200; PMCID: PMC3532882.
2: Zhu HY, Guo HF, Hou HL, Liu YJ, Sheng SL, Zhou JN. Increased expression of the Nogo receptor in the hippocampus and its relation to the **neuropathology in Alzheimer's disease**. Hum Pathol. 2007 Mar;38(3):426-34. doi: 10.1016/j.humpath.2006.09.010. Epub 2006 Dec 22. PMID: 17188332.
3: VanGuilder Starkey HD, Bixler GV, Sonntag WE, Freeman WM. Expression of NgR1-antagonizing proteins decreases with **aging and cognitive decline** in rat hippocampus. Cell Mol Neurobiol. 2013 May;33(4):483-8. doi:

10.1007/s10571-013-9929-4. Epub 2013 Mar 24. PMID: 23525710; PMCID: PMC3651369.
4: Li X, Fu QL, Jing XL, Liao XX, Zeng AH, Xiong Y, Liao XX. Myelin-associated glycoprotein inhibits the **neuronal differentiation of neural progenitors**. Neuroreport. 2009 May 6;20(7):708-12. doi: 10.1097/WNR.0b013e32832aa942. PMID: 19349915.
5: Tong J, Liu W, Wang X, Han X, Hyrien O, Samadani U, Smith DH, Huang JH. Inhibition of Nogo-66 receptor 1 enhances **recovery of cognitive function** after traumatic brain injury in mice. J Neurotrauma. 2013 Feb 15;30(4):247-58. doi: 10.1089/neu.2012.2493. Epub 2013 Feb 4. PMID: 22967270; PMCID: PMC3579383.

Rps6kb1
1: Venkatasubramani JP, Subramanyam P, Pal R, Reddy BK, Srinivasan DJ, Chattarji S, Iossifov I, Klann E, Bhattacharya A. N-terminal variant Asp14Asn of the human p70 S6 Kinase 1 enhances translational signaling causing different effects in **developing and mature neuronal cells**. Neurobiol Learn Mem. 2020 May;171:107203.
doi: 10.1016/j.nlm.2020.107203. Epub 2020 Mar 5. PMID: 32147585.

Trpc4
1: Zechel S, Werner S, von Bohlen Und Halbach O. Distribution of TRPC4 in **developing and adult murine brain**. Cell Tissue Res. 2007 Jun;328(3):651-6. doi: 10.1007/s00441-007-0388-4. Epub 2007 Mar 8. PMID: 17345099.
2: Arboit A, Reboreda A, Yoshida M. Involvement of TRPC4 and 5 Channels in **Persistent Firing in Hippocampal CA1 Pyramidal Cells**. Cells. 2020 Feb 5;9(2):365. doi: 10.3390/cells9020365. PMID: 32033274; PMCID: PMC7072216.
3: Bröker-Lai J, Kollewe A, Schindeldecker B, Pohle J, Nguyen Chi V, Mathar I, Guzman R, Schwarz Y, Lai A, Weißgerber P, Schwegler H, Dietrich A, Both M, Sprengel R, Draguhn A, Köhr G, Fakler B, Flockerzi V, Bruns D, Freichel M. Heteromeric channels formed by TRPC1, TRPC4 and TRPC5 define **hippocampal synaptic transmission and working memory**. EMBO J. 2017 Sep 15;36(18):2770-2789. doi: 10.15252/embj.201696369. Epub 2017 Aug 8. PMID: 28790178; PMCID: PMC5599800.

Tcf4
(34 hits) e.g.
1: Kennedy AJ, Rahn EJ, Paulukaitis BS, Savell KE, Kordasiewicz HB, Wang J,Lewis JW, Posey J, Strange SK, Guzman-Karlsson MC, Phillips SE, Decker K, Motley ST, Swayze EE, Ecker DJ, Michael TP, Day JJ, Sweatt JD. Tcf4 Regulates **Synaptic Plasticity, DNA Methylation, and Memory Function**. Cell Rep. 2016 Sep 6;16(10):2666-2685. doi: 10.1016/j.celrep.2016.08.004. Epub 2016 Aug 25. PMID: 27568567; PMCID: PMC5710002.
2: Crux S, Herms J, Dorostkar MM. Tcf4 **regulates dendritic spine density** and morphology in the adult brain. PLoS One. 2018 Jun 22;13(6):e0199359. doi: 10.1371/journal.pone.0199359. PMID: 29933371; PMCID: PMC6014661.
3: Schoof M, Hellwig M, Harrison L, Holdhof D, Lauffer MC, Niesen J, Virdi S, Indenbirken D, Schüller U. The basic helix-loop-helix transcription factor TCF4 **impacts brain architecture as well as neuronal morphology and differentiation**. Eur J Neurosci. 2020 Jun;51(11):2219-2235. doi: 10.1111/ejn.14674. Epub 2020 Jan 24. PMID: 31919899.
4: Brzózka MM, Rossner MJ. Deficits in trace fear memory in a mouse model of the **schizophrenia risk gene TCF4**. Behav Brain Res. 2013 Jan 15;237:348-56. doi: 10.1016/j.bbr.2012.10.001. Epub 2012 Oct 13. PMID: 23069005.

Sorcs3
1: Christiansen GB, Andersen KH, Riis S, Nykjaer A, Bolcho U, Jensen MS, Holm MM. The sorting receptor SorCS3 is a **stronger regulator of glutamate receptor** functions compared to GABAergic mechanisms in the hippocampus. Hippocampus. 2017 Mar;27(3):235-248. doi: 10.1002/hipo.22689. Epub 2017 Feb 3. PMID: 27935149.
2: Breiderhoff T, Christiansen GB, Pallesen LT, Vaegter C, Nykjaer A, Holm MM, Glerup S, Willnow TE. Sortilin-related receptor **SORCS3 is a postsynaptic modulator of synaptic depression and fear extinction**. PLoS One. 2013 Sep 19;8(9):e75006. doi: 10.1371/journal.pone.0075006. PMID: 24069373; PMCID: PMC3777878.
3: Hermey G, Plath N, Hübner CA, Kuhl D, Schaller HC, Hermans-Borgmeyer I. The three sorCS genes are differentially expressed and regulated **by synaptic activity**. J Neurochem. 2004 Mar;88(6):1470-6. doi: 10.1046/j.1471-4159.2004.02286.x. PMID: 15009648.

Zeb2
1: Epifanova E, Babaev A, Newman AG, Tarabykin V. Role of Zeb2/Sip1 in **neuronal development**. Brain Res. 2019 Feb 15;1705:24-31. doi: 10.1016/j.brainres.2018.09.034. Epub 2018 Sep 25. PMID: 30266271.
2: Hegarty SV, Sullivan AM, O'Keeffe GW. Zeb2: A multifunctional **regulator** of nervous system development. Prog Neurobiol. 2015 Sep;132:81-95. doi: 10.1016/j.pneurobio.2015.07.001. Epub 2015 Jul 18. PMID: 26193487.

Table S3

Functional enrichment analysis with g:Profiler for protein groups exclusively identified in the CA1 region. GO:MF gene ontology term for molecular function; GO:CC gene ontology for cellular component; GO:BP gene ontology for biological process; the specific GO term ID is given. Rich factor is the ratio of differentially expressed protein number annotated in this pathway term (intersections size) to all protein number annotated (term size). The p-value represents the probability of the observed intersection plus probabilities of all larger, more extreme intersections.

source	term_id	term_name	intersection size	rich factor	adjusted p_value	negative log10 of adjusted p_value	term_size
GO:MF	GO:0005488	binding	346	0.0214	2.80E-15	14.55	16182
GO:MF	GO:0140096	catalytic activity; acting on a protein	72	0.0311	5.49E-05	4.26	2313
GO:MF	GO:0019199	transmembrane receptor protein kinase activity	9	0.1125	3.52E-03	2.45	80
GO:MF	GO:0032555	purine ribonucleotide binding	57	0.0297	5.68E-03	2.25	1918
GO:MF	GO:0005198	structural molecule activity	34	0.0366	7.05E-03	2.15	928
GO:MF	GO:0008289	lipid binding	32	0.0368	1.18E-02	1.93	870
GO:CC	GO:0110165	cellular anatomical entity	405	0.0177	2.19E-16	15.66	22824
GO:CC	GO:0030054	cell junction	86	0.0402	1.92E-14	13.72	2140
GO:CC	GO:0045202	synapse	66	0.0436	3.46E-12	11.46	1515
GO:CC	GO:0043005	neuron projection	64	0.0394	8.49E-10	9.07	1626
GO:CC	GO:0036477	somatodendritic compartment	41	0.0369	5.24E-05	4.28	1110
GO:CC	GO:0097447	dendritic tree	31	0.0390	5.76E-04	3.24	795
GO:BP	GO:0032502	developmental process	174	0.0256	1.13E-10	9.95	6802
GO:BP	GO:0048856	anatomical structure development	163	0.0263	1.15E-10	9.94	6197
GO:BP	GO:0050808	synapse organization	27	0.0553	2.72E-05	4.57	488
GO:BP	GO:0010646	regulation of cell communication	94	0.0265	1.38E-04	3.86	3547
GO:BP	GO:0048812	neuron projection morphogenesis	30	0.0439	7.11E-04	3.15	684
GO:BP	GO:0007399	nervous system development	71	0.0281	1.04E-03	2.98	2530

Table S4

IPA information for specific categories

IPA analysis - categories linked to disease and functional annotation were listed by a p-value of at least p>0,05. The number of participating molecules out of our list is provided.

Categories	Diseases or Functions Annotation	p-value	# Molecules
Cell Morphology,Cellular Assembly and Organization,Cellular Function and Maintenance	Formation of cellular protrusions	1,37E-03	45
Cellular Development,Cellular Growth and Proliferation,Nervous System Development and Function,Tissue Development	Development of neural cells	3,44E-03	51
Nervous System Development and Function,Neurological Disease,Organ Morphology,Organismal Development,Organismal Injury and Abnormalities	Abnormal morphology of hippocampus	4,18E-03	9
Cell-To-Cell Signaling and Interaction,Cellular Assembly and Organization,Nervous System Development and Function,Tissue Morphology	Density of synapse	6,32E-03	5
Nervous System Development and Function	Development of central nervous system	8,92E-03	38
Cell Morphology,Cellular Assembly and Organization,Cellular Development,Cellular Function and Maintenance,Cellular Growth and Proliferation,Embryonic Development,Nervous System Development and Function,Organismal Development,Tissue Development	Dendritic growth/branching	1,06E-02	20
Cell Morphology,Cellular Assembly and Organization,Cellular Development,Cellular Function and Maintenance,Cellular Growth and Proliferation,Nervous System Development and Function,Organismal Development,Tissue Development	Axonogenesis	2,45E-02	14
Cell Morphology,Nervous System Development and Function,Tissue Morphology	Morphology of neurites	3,84E-02	16

Table S5

A LC-MS/MS parameters (data independent mode; quantitative data)

Data independent analyses (DIA)	
reversed phase liquid chromatography	**Ultimate 3000 RSLC (Thermo Scientific)**
Trap column	75 µm inner diameter, packed with 3 µm C18 particles (Acclaim PepMap100, Thermo Scientific)
Analytical column	75 µm inner diameter, packed with 2.6 µm C18 particles (Accucore, 25 cm, Thermo Scientific)
Flow rate	300 nl/min
column oven temperature	40°C
buffer system	binary buffer system consisting of 0.1% acetic acid in HPLC-grade water (buffer A) and 100% ACN in 0.1% acetic acid (buffer B)
gradient	gradient of buffer B: 2min 2% to 5 %, 8min 5%, 120min 5% to 25%, 5min 25 to 40%, 2 min 40% to 90%, 5 min 90%, 3 min 90% to 2%, 5 min 2%
Mass spectrometer	**Q Exactive HF**
operation mode	data-independent
electrospray	Nanospray Flex Ion Source
Full MS	
MS scan resolution	60,000
AGC target	5e6
maximum ion injection time for the MS scan	200 ms
Scan range	333 to 1650 *m/z*
Spectra data type	profile
dd-MS2	
Resolution	30,000
MS/MS AGC target	3e6
maximum ion injection time for the MS/MS scans	auto
Spectra data type	profile
selection for MS/MS	1
isolation window	56 windows *m/z* 13
Fixed first mass	200
dissociation mode	higher energy collisional dissociation (HCD)
normalized collision energy	stepped, 27.5
dissociation mode	HCD

B Spectronaut parameters for peptide/protein identification and intensity extraction

Spectronaut 17.1.221229.55965	
Computer Name: AGVOE-SPECTRONA	
User Domain Name: AGVOE-SPECTRONA	
User Name: spectronaut	
Analysis Mode: UI	
Analysis Type: directDIA	
Settings Used:	
Pulsar Search\Peptides	
Toggle N-terminal M:	True
Min Peptide Length:	7
Max Peptide Length:	52
Missed Cleavages:	2
Digest Type:	Specific
Enzymes / Cleavage Rules:	Trypsin/P
directDIA workflow:	DirectDIA+(Deep)
Pulsar Search\Labeling	
Channels:	
Channel 1:	False
Channel 2:	False
Channel 3:	False
DIA Analysis\Data Extraction	
MS1 Mass Tolerance Strategy:	Dynamic
Correction Factor:	1
MS2 Mass Tolerance Strategy:	Dynamic
Correction Factor:	1
Intensity Extraction MS1:	Maximum Intensity
Intensity Extraction MS2:	Maximum Intensity
DIA Analysis\XIC Extraction	
XIC IM Extraction Window:	Dynamic
Correction Factor:	1
XIC RT Extraction Window:	Dynamic
Correction Factor:	1
Pulsar Search\Modifications	
Max Variable Modifications:	5
Database	
Original File:	mouse_uniprot_04_2022.fasta
Select Modifications:	
Fixed Modifications::	Carbamidomethyl (C)
Variable Modifications: :	Acetyl (Protein N-term), Oxidation (M)
DIA Analysis\Calibration	
MS1 Mass Tolerance Strategy:	System Default
MS2 Mass Tolerance Strategy:	System Default
Precision iRT:	True

iRT <-> RT Regression Type:	Local (Non-Linear) Regression
Exclude Deamidated Peptides:	True
MZ Extraction Strategy:	Maximum Intensity
Allow source specific iRT Calibration:	True
DIA Analysis\Identification	
Generate Decoys:	True
Decoy Limit Strategy:	Dynamic
Library Size Fraction:	0.1
Decoy Method:	Mutated
Preferred Fragment Source:	NN Predicted Fragments
Machine Learning:	Per Run
Exclude Duplicate Assays:	True
Precursor PEP Cutoff:	0.2
Protein Qvalue Cutoff (Experiment):	0.01
Protein Qvalue Cutoff (Run):	0.05
Exclude Single Hit Proteins:	False
Pvalue Estimator:	Kernel Density Estimator
Precursor Qvalue Cutoff:	0.001
Single Hit Definition:	By Stripped Sequence
DIA Analysis\Quantification	
Interference Correction:	True
MS1 Min:	2
MS2 Min:	3
Exclude All Multi-Channel Interferences:	True
Only Identified Peptides:	True
Protein LFQ Method:	Automatic
Major (Protein) Grouping:	by Protein Group Id
Minor (Peptide) Grouping:	by Stripped Sequence
Minor Group Top N:	False
Minor Group Quantity:	Sum precursor quantity
Major Group Top N:	True
Min:	2
Max:	3
Major Group Quantity:	Mean peptide quantity
Quantity MS-Level:	MS2
Quantity Type:	Area
Proteotypicity Filter:	None (set to proteotypic by in-house analysis R pipeline)
Data Filtering:	Qvalue sparse
Fraction:	NA
Imputing Strategy:	No Imputing
Cross Run Normalization:	True (local)
DIA Analysis\PTM Workflow	
PTM Localization:	True
Probability Cutoff:	0.75
PTM Analysis:	True
Multiplicity:	True

Run Clustering:	False
PTM Consolidation:	Sum
Flanking Region:	7
DIA Analysis\Workflow	
MS2 DeMultiplexing:	Automatic
Run Limit for directDIA Library:	-1
Method Evaluation:	False
Profiling Strategy:	iRT Profiling
Profiling Row Selection:	Minimum Qvalue Row Selection
Qvalue Threshold:	0.001
Profiling Target Selection:	Profile only non-identfied Precursor
Identification Criterion:	Qvalue
Threshold:	0.001
Carry-over exact Peak Boundaries:	False
Unify Peptide Peaks Strategy:	Select corresponding Peak
DIA Analysis\Protein Inference	
Protein Inference Workflow:	Automatic
Inference Algorithm:	IDPicker
DIA Analysis\Post Analysis	
Calculate Sample Correlation Matrix:	True
Calculate Explained TIC:	Quick
Differential Abundance Grouping:	Major Group (Quantification Settings)
Smallest Quantitative Unit:	Precursor Ion (Quantification Settings)
Use All MS-Level Quantities:	False
Differential Abundance Testing:	Paired t-test
Group-Wise Testing Correction:	False
Run Clustering:	True
Distance Metric:	Manhattan Distance
Linkage Strategy:	Ward's Method
Z-score transformation:	False
Order Runs by Clustering:	True
DIA Analysis\Pipeline Mode	
Post Analysis Reports:	
Scoring Histograms:	True
Data Completeness Bar Chart:	True
Run Identifications Bar Chart:	True
CV Density Line Chart:	True
CVs Below X Bar Chart:	True
Generate SNE File:	True
Store Iontraces in SNE:	False
Report Schema:	C_FunGene_complex (Normal)
Reporting Unit:	Across Experiment
Pulsar Search\Identification	
Peptide FDR:	0.01
Protein Group FDR:	0.01
PSM FDR:	0.01

Pulsar Search\Tolerances	
Tolerance Parameters:	
Thermo Orbitrap:	
Calibration Search:	Dynamic
MS1 Correction Factor:	1
MS2 Correction Factor:	1
Main Search:	Dynamic
MS1 Correction Factor:	1
MS2 Correction Factor:	1
TOF:	
Calibration Search:	Dynamic
MS1 Correction Factor:	1
MS2 Correction Factor:	1
Main Search:	Dynamic
MS1 Correction Factor:	1
MS2 Correction Factor:	1
Thermo IonTrap:	
Calibration Search:	Dynamic
MS1 Correction Factor:	1
MS2 Correction Factor:	1
Main Search:	Dynamic
MS1 Correction Factor:	1
MS2 Correction Factor:	1
Pulsar Search\Workflow	
Use DNN Predicted Ion Mobility:	Auto
Fragment Ion Selection Strategy:	Intensity Based
In-Silico Generate Missing Channels:	False
Pulsar Search\Result Filters	
Precursors:	
Best N Fragments per Peptide:	True
Min:	6
Max:	10
Channel Count:	False
Modifications:	None
Amino Acids:	False
Best N Peptides per Protein Group:	False
FASTA Matched:	False
Missed Cleavage:	False
Peptide Charge:	False
Proteotypicity:	False
Fragment Ions:	
m/z :	True
Min:	300
Max:	1800
Ion Charge:	False
Ion Loss Type:	False

Ion Type:	False
Ion AA Length:	True
N:	3
Relative Intensity:	True
Min:	5

Figure S1

Removal of the mounting medium and tissue sampling.

Glass slides with the embedded sections were removed from the refrigerator and were transferred into xylene (between 24 – 72 h). Next, the slides were transferred into 96% ethanol for about 1 h (due the treatment with xylene, some air bubbles might occur (**1 a**)). Using a razorblade, the coverslip was gingerly lifted in one corner (**1 b**). The slide was transferred again for 1 hour in 96% ethanol. Thereafter, the coverslip was removed (**1 c**). The slide with the sections was transferred to different ethanol solutions in a descending order (96%, 70%, 50%, 20%; each for about 5 min. on a shaker). Using a scalpel the desired piece of tissue was cut of the brain section (**1 d**) and was transferred into the lid of an Eppendorf tube containing the solution necessary for further processing (**1 e, f**). Sampling was continued as described until the region of interest has been taken from four sections on both hemispheres.

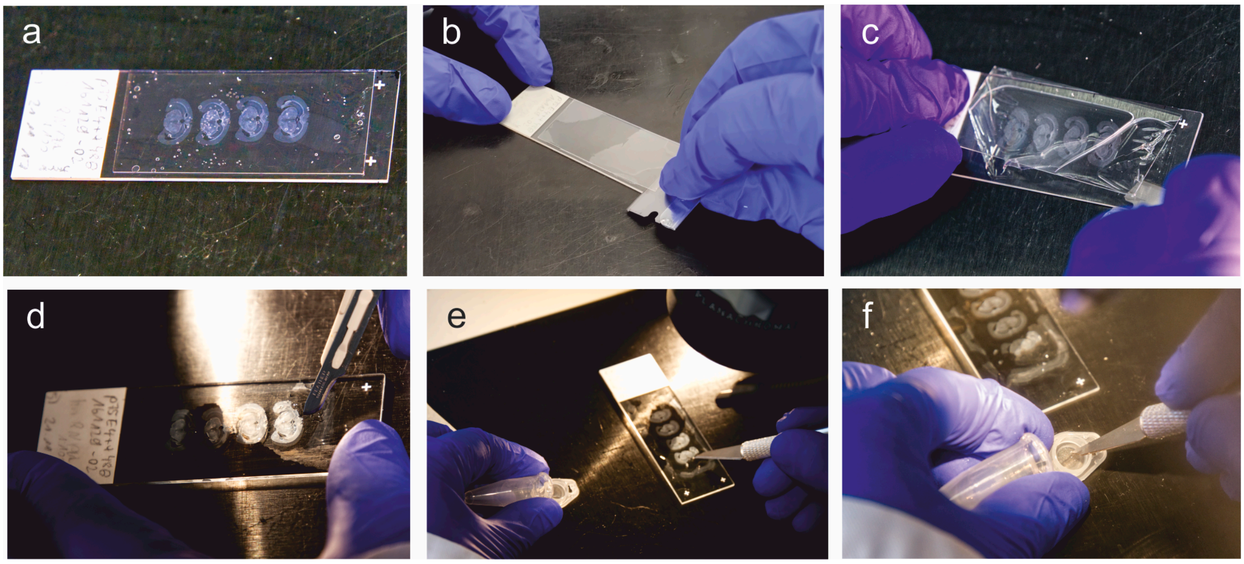

The hippocampus can clearly be identified due to the DAPI counterstaining that visualizes nuclear DNA (in blue).

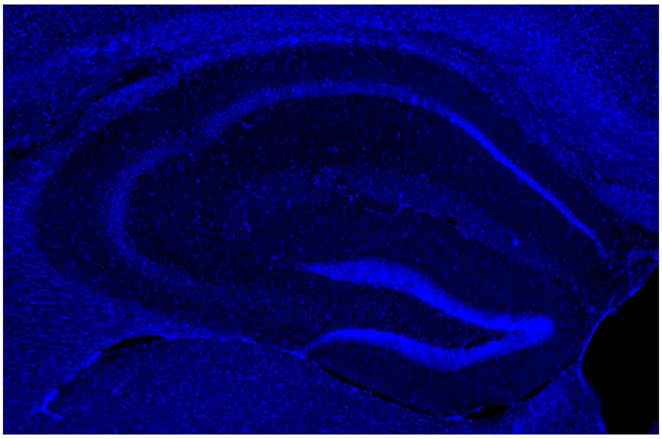

Figure S2

Protein intensities of 27 hippocampal proteins.

Gene names of proteins exclusively identified of the CA1 region extracts are provided at the y-axis**.** x-axis represents the protein intensities as mean values with SEM (n=9).

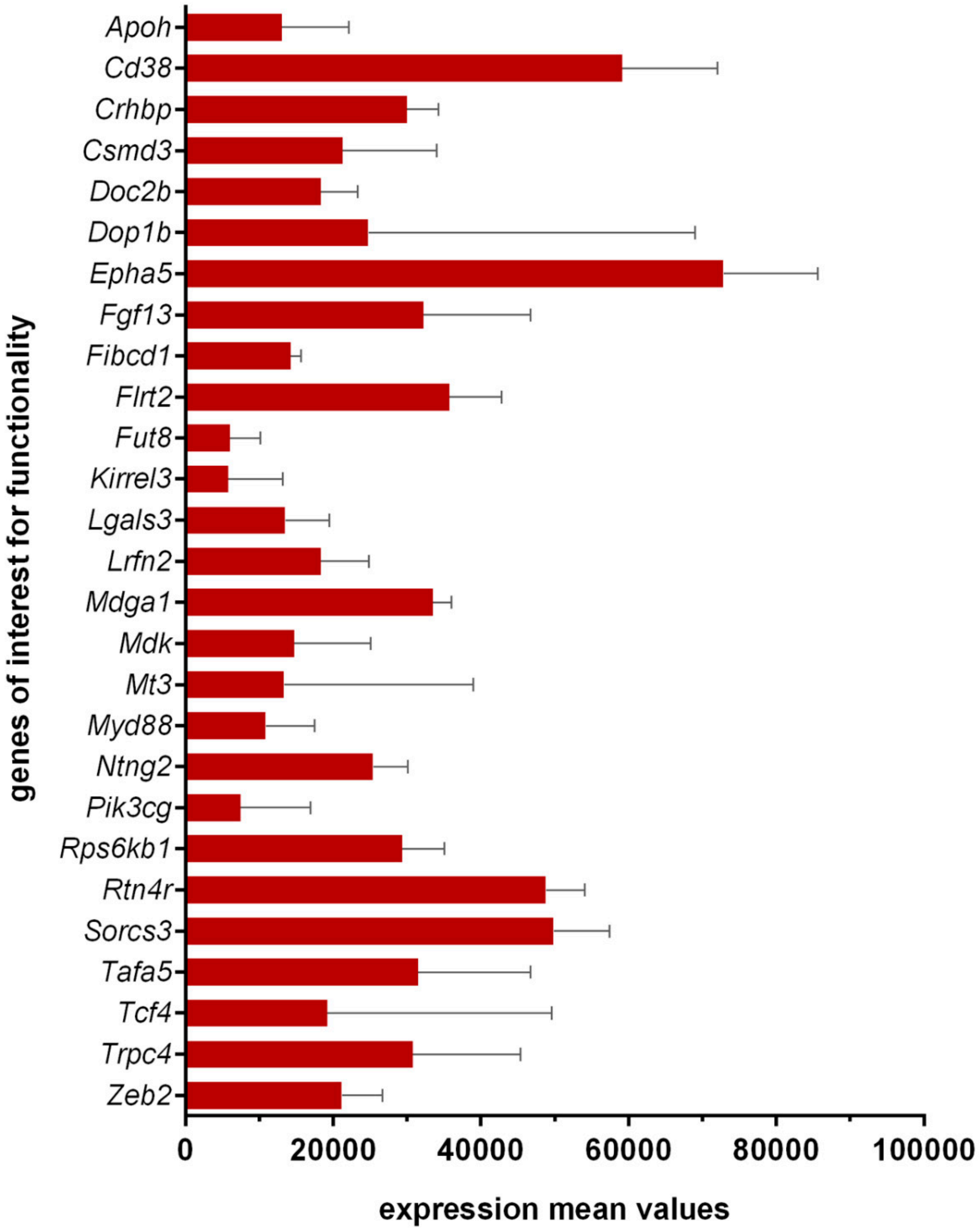

Figure S3

Protein-protein-interaction network of proteins presented in Table 2.

Proteins involved in „Axon guidance“ are labelled in blue and proteins involved in G-beta-signalling are labeld in red. Furthermore, sodium and calcium channels belong to the network.

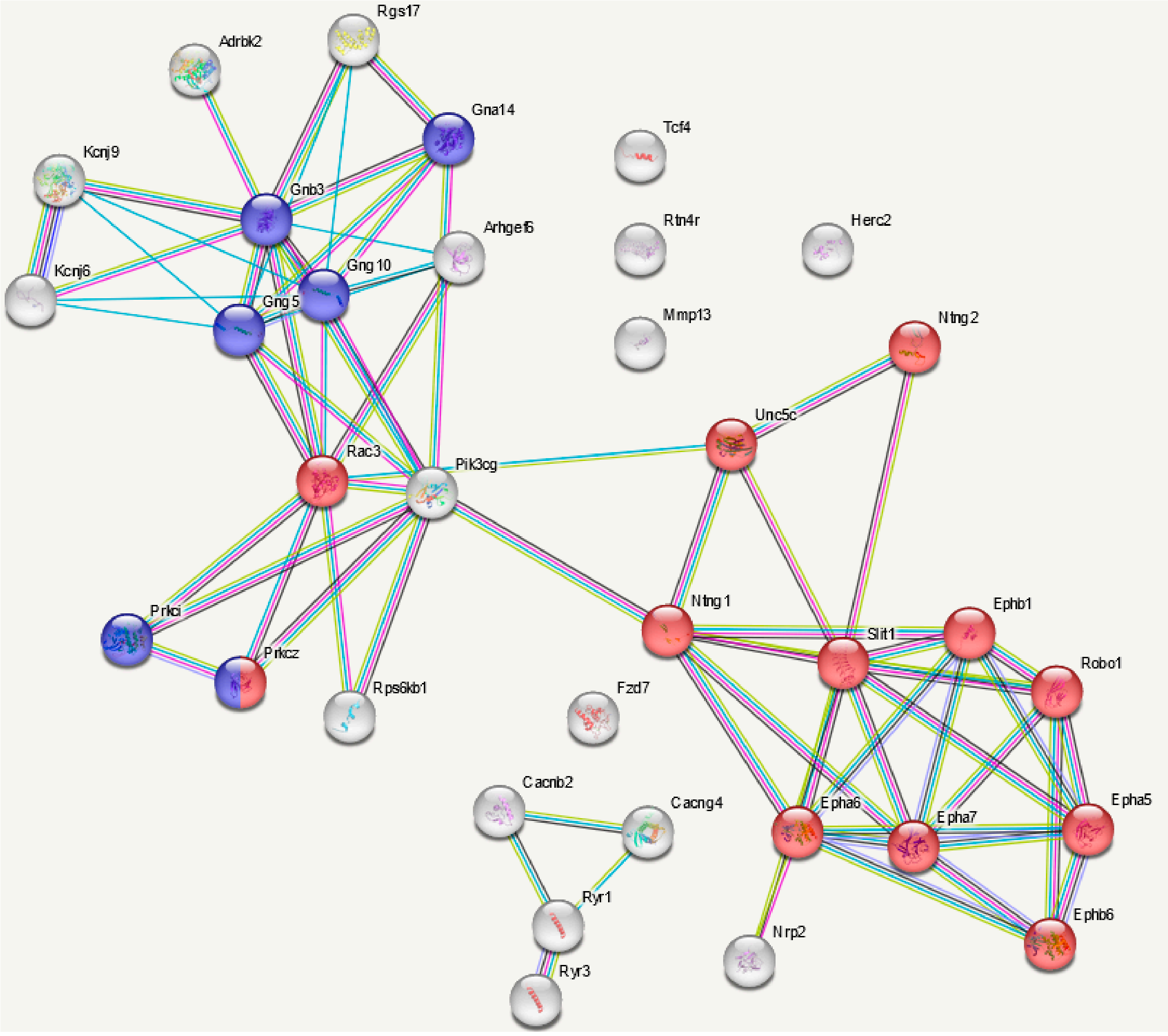

Figure S4

IPA networks.

A and B (same name, different molecules included: Graphic representation of the network “Cell Death and Survival, Neurological Disease, Organismal Injury and Abnormalities”; C: Graphic representation of the network “Cell Death and Survival, Free Radical Scavenging, Organismal Injury and Abnormalities”

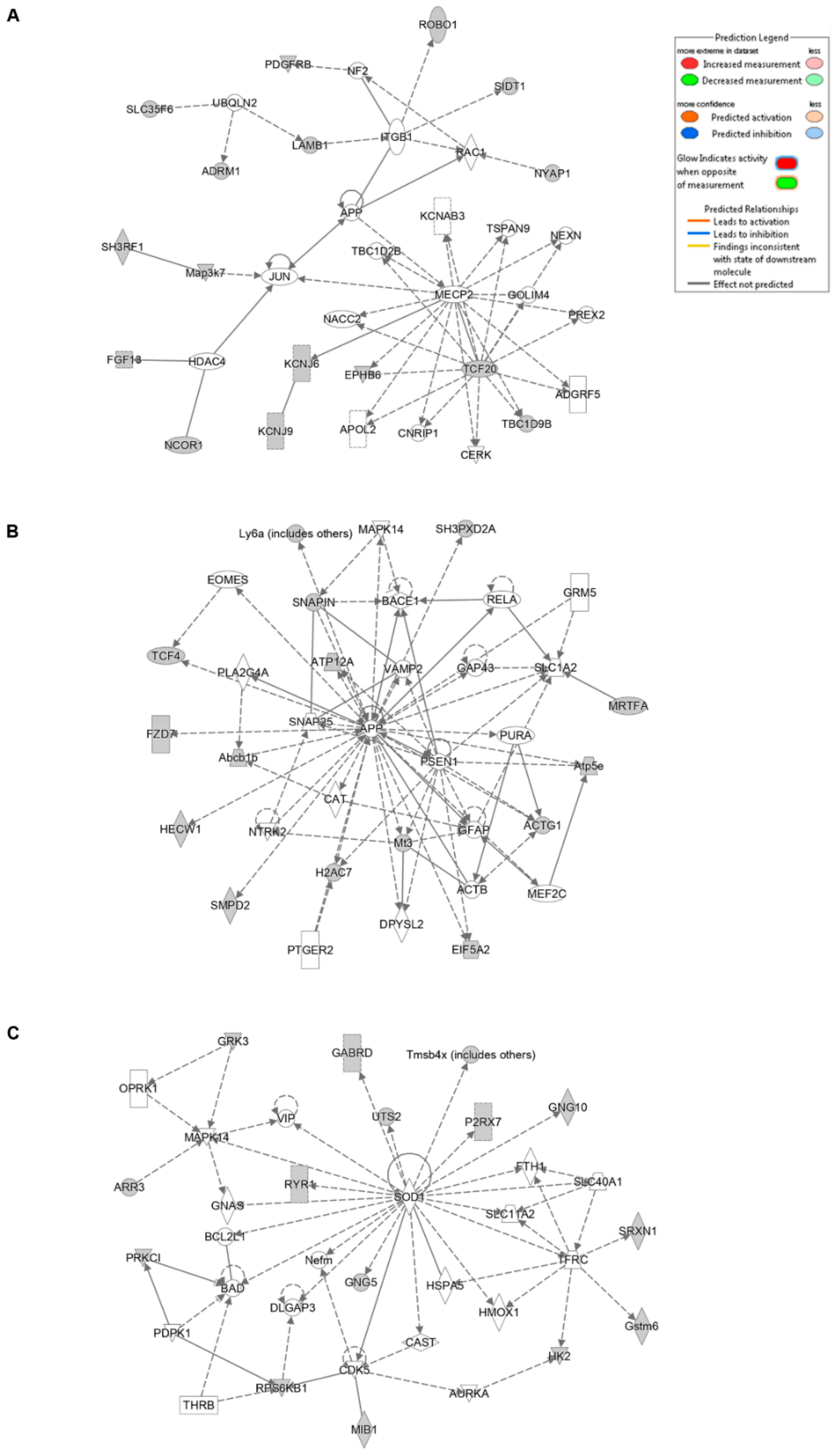

Supplement: Supplementary file 1 [file ijms-24-11308-s001.zip › 20230707_ijms-2482729_method paper_Supplements_SV_rev2.pdf]
